# Progressive Blood–Brain Barrier Disruption in Sleep-Restricted Young Mice: Cellular Senescence and Neuroinflammation Crosstalk

**DOI:** 10.1007/s11064-025-04510-y

**Published:** 2025-08-18

**Authors:** Jessica J. Avilez-Avilez, Jesús Enrique García-Aviles, Ricardo Jair Ramírez-Carreto, Verónica Salas-Venegas, Mara A. Guzmán-Ruiz, Fernanda Medina-Flores, Mina Königsberg, Anahí Chavarría, Beatriz Gómez-González

**Affiliations:** 1 Posgrado en Biología Experimental, DCBS, Universidad Autónoma Metropolitana-Iztapalapa, Mexico City, Mexico; 2 Área de Neurociencias, Departamento de la Biología de la Reproducción, Universidad Autónoma Metropolitana-Iztapalapa, Mexico City, Mexico; 3 Departamento de Fisiología, Facultad de Medicina, Universidad Nacional Autónoma de México, Mexico City, Mexico; 4 Facultad de Medicina, Unidad de Investigación en Medicina Experimental “Dr. Ruy Pérez Tamayo”, Universidad Nacional Autónoma de México, Mexico City, Mexico; 5 Programa de Doctorado en Ciencias Biomédicas, Universidad Nacional Autónoma de México, Mexico City, Mexico; 6 Programa de Becas Postdoctorales, Dirección General de Asuntos del Personal Académico, Universidad Nacional Autónoma de México, Mexico City, Mexico; 7 Cell Biology Department, Emory University, Atlanta, GA USA; 8 Departamento de Ciencias de la Salud, Universidad Autónoma Metropolitana-Iztapalapa, Mexico City, Mexico

**Keywords:** Astrogliosis, Blood–brain barrier, Cellular senescence, Neuroinflammation, Sleep restriction

## Abstract

Sleep loss promotes a chronic low-grade inflammatory status with increased levels of inflammatory cytokines. Sleep loss also induces low-grade neuroinflammation characterized by glial reactivity and blood–brain barrier (BBB) dysfunction, as evidenced by BBB hyperpermeability and tight junction disassembly. Additionally, it raises molecules related to the senescence-associated secretory phenotype (SASP) in aged subjects, suggesting an increase in senescent cells. Here, we assessed the impact of sleep restriction on cellular senescence, neuroinflammation, and BBB function in the cerebral cortex and hippocampus of young male C57BL/6 mice. Sleep restriction induced a progressive increase in BBB permeability after 3, 5, and 10 days, along with a higher expression of the astroglial marker, the glial fibrillary acidic protein (GFAP), and the expression of the C3 complement component. The pro-inflammatory cytokines tumor necrosis factor-α (TNF-α), interleukin-1 beta (IL-1β), and interleukin-6 (IL-6) increased in a region-dependent form. Furthermore, the progressive increase of the senescence markers β-galactosidase and p21 observed in both brain regions was accompanied by a neurotoxic astroglial response. Our data suggest that sleep restriction promotes cellular senescence in the cerebral cortex and hippocampus of young mice.

**Graphical Abstract:**

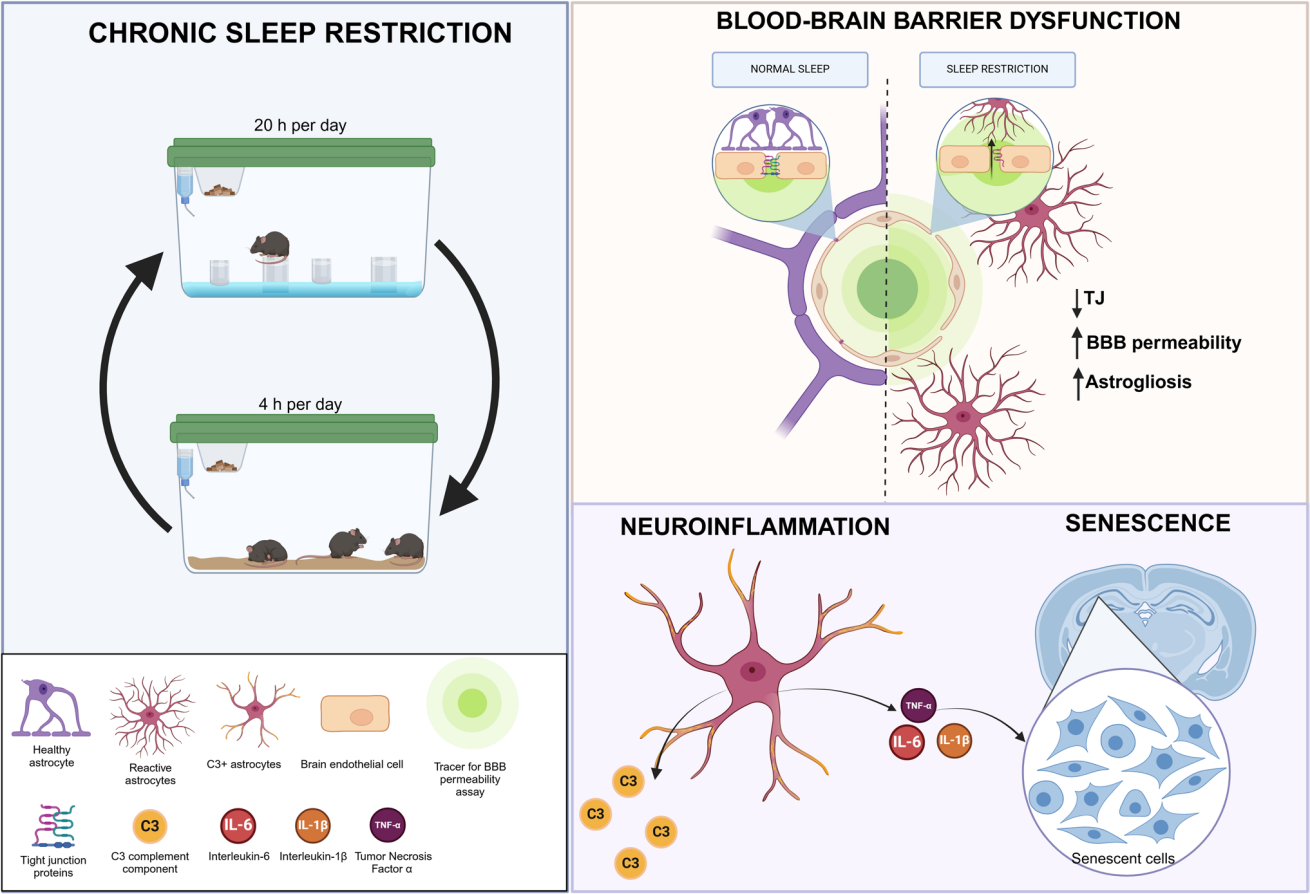

**Supplementary Information:**

The online version contains supplementary material available at 10.1007/s11064-025-04510-y.

## Introduction

Sleep is a fundamental activity that preserves life in organisms, as it maintains homeostasis both centrally and peripherally. Poor sleep quality has been linked to premature aging [1] and neuroinflammation [2].

Sleep loss promotes the reactivity of astroglia and microglia [3]. Astrogliosis may contribute to neuroinflammation, as astrocytes are among the primary producers of tumor necrosis factor-α (TNF-α) [4] and of complement components, such as C3, in the central nervous system (CNS) [5].

Increased levels of C3 protein are associated with neuroinflammation and the activation of microvascular endothelial cells [6, 7], as reported following sleep deprivation [8]. In aged animals, C3 binds to C3aR in endothelial cells, leading to a reduced trans-endothelial electric resistance (TEER) and raised BBB permeability [9].

It is known that ten days of sleep restriction induces elevated BBB permeability by decreasing the expression of the tight junction proteins claudin-5 (cldn-5), occludin, and zonula occludens-1 (ZO-1) [2, 10], promoting pericyte detachment from the capillary wall [11]. Chronic sleep restriction also increased the serum levels of the proinflammatory cytokines TNF-α and IFNγ [2]. In the CNS, this condition led to elevations in phosphorylated-NFkB, matrix metalloproteinase-9 (MMP-9), and the A_2A_ adenosine receptor [11].

Sleep loss has also been associated with cellular senescence in aging models [12, 13]. Cellular senescence is a permanent state of growth arrest triggered by stress, in which cells stop dividing but remain metabolically active. Senescence can be induced by telomere shortening due to cell replication (replicative senescence) or in response to cell damage, including oxidative stress and the inflammatory microenvironment (stress-induced senescence) [14]. The increases in cyclin-dependent kinase (CDK) inhibitors, such as p16 and p21, as well as lysosomal changes (indicated by the β-galactosidase enzyme), are considered markers of cellular senescence. Its key feature is the secretion of a set of inflammatory molecules known as the senescence-associated secretory phenotype (SASP), which involves IL-1α, IL-8, IL-1β, and IL-6, thereby contributing to neuroinflammation [15, 16].

Sleep loss also induces senescence in the CNS, which is cumulative and contributes to BBB dysfunction and neuroinflammation.

Our results showed that sleep restriction progressively increased BBB permeability in the cerebral cortex and hippocampus from days 3 to 10 in young mice. Over the 10 days of sleep loss, a progressive increase in the expression of the inflammatory marker glial fibrillary acidic protein (GFAP) was observed, accompanied by a rise in the expression of proinflammatory cytokines TNFα, IL-6, and IL1-β at 10 days of sleep restriction. Complementarily, a decrease in the astroglial neuroprotective response marker S100a10 was observed, along with an increase in senescent markers p16 and β-galactosidase. These findings suggest that sleep loss induces astrocyte reactivity accompanied by cellular senescence and that both phenotypes contribute to neuroinflammation in young mice.

## Methods

### Animals

Male C57BL/6 mice *(Mus musculus)* (20–27 g), 8–10 weeks old, were used in this study. Animals were provided by the vivarium of the Unidad de Medicina Experimental, Facultad de Medicina, Universidad Nacional Autónoma de México. Mice were housed 3–6 per cage in our laboratory vivarium under a 12 h light/dark cycle (lights on at 2 am) at 20–22 °C, with water and food ad libitum for the days the experiment lasted, including 15 days of adaptation. All animal procedures were strictly carried out by the Mexican Official Ethics Standard NOM-062-ZOO-1999 and the Standard for the Disposal of Biological Waste (NOM-087-ECOL-1995), and were approved by the Academic Ethics Committee of the Biological Sciences Division at the Universidad Autónoma Metropolitana, Unidad Iztapalapa.

### Experimental Groups

Mice were randomly assigned to four experimental conditions: 3-day sleep-restricted group (SR3), 5-day sleep-restricted group (SR5), 10-day sleep-restricted group (SR10), and controls, which normally slept in their home cages (CON).

### Sleep Restriction Model

Sleep restriction was carried out using the modified multiple platform method (MMPM), which has been previously reported and validated by EEG in our group and effectively increases the wake time [2, 17]. MMPM included two platforms (diameter, 2.5–3 cm) surrounded by water (1 cm deep) in an acrylic cage. Mice were individually placed in each cage. The sleep restriction protocol consisted of 20 h of sleep restriction daily with 4 h of sleep opportunity during the last 4 h of the light phase, when the mice were moved to their social group cages (to avoid social isolation stress), where they were allowed to sleep.

### Blood–Brain Barrier Permeability Assay

At the end of the sleep restriction period, animals were anesthetized (n = 5 per group) with sodium pentobarbital lethal dose (ip. 0.063 g/kg body weight), followed by a cocktail of Evans blue (1 mg/mL) and fluorescein-sodium (Na-F; 10 mg/mL) administration (0.1 mL per 20 g of body weight) in the left heart ventricle. The cocktail tracers circulated for 3 min; at the end of this period, subjects were perfused with 0.9% saline solution for 3 min. The cerebral cortex and hippocampus were dissected. Brain samples were weighed and homogenized with 200 µL phosphate-buffered solution (PBS) 1X and 200 µL of absolute methanol. Homogenates were centrifuged at 13,500 rpm for 10 min. After that, 100 µl of the supernatant was collected and placed in a 96-well plate using an ELISA plate reader, as previously reported [2, 11].

### Western Blot

Animals were euthanized by decapitation. Brains were obtained to evaluate protein expression in intact controls or sleep-restricted mice during 3, 5, or 10 days (n = 4–6 per group). The cerebral cortex and hippocampus were dissected, frozen, and stored at -80 °C until processing. Brain tissue was homogenized with a lysis buffer (200 µL) that contained a protease inhibitor cocktail (ROCHE 11836153001) and centrifuged at 13,500 rpm for 10 min at 4 °C. Protein concentration was determined using the Bradford assay (BioRad, 500–0006).

Proteins (100 µg) were resolved using a denaturing 10% SDS-PAGE electrophoresis and transferred to PVDF membranes (duplicated). Membranes were blocked with 10% non-fat milk in PBS-Tween 0.1% for 30 min at room temperature and incubated overnight at 4 °C using the following primary antibodies (online resource 1): anti-claudin-5 (Biorbyt, orb160461, 1:500), anti-ZO-1 (Life technologies 402,200; 1:1000), anti-GFAP (Abcam, ab4648, 1:1000), anti-C3 (Abcam, ab200999; 1:1000), anti-S100a10 (Novus Biologicals, NBP1 89,370; 1:1000), anti-p21 (Santa Cruz Biotechnology, sc-6246, 1:1000), anti-β-galactosidase (Santa Cruz Biotechnology, sc-65670, 1:1000). After three washes, PVDF membranes were incubated with biotinylated anti-mouse (Vector laboratories, BA-9200) or biotinylated anti-rabbit (Vector laboratories, BA-1000) secondary antibodies (1:2500). After three more washes, membranes were incubated with an ABC kit (Vectastain PK-6100) for 30 min. Finally, membranes were revealed with a chemiluminescence detection system (Immobilon Western WBKLS0500). Membranes were stained with Ponceau red. Images from PVDF membranes were acquired using C-Digit (LI-COR iS; online resource 2). Blots and Ponceau optical densities were obtained with ImageStudio software, Version 5.1. The entire line of Ponceau red (optical density) was used as a loading control and for normalization. Normalization was performed by obtaining a protein-to-Ponceau ratio; subsequently, the fold change from the control was calculated (CON = 1). Values greater than 1 indicate an increased effect, while values below 1 indicate a reduction in the impact compared to the CON [18].

### Enzyme-Linked Immunosorbent Assay (ELISA)

The cerebral cortex and hippocampus were dissected from CON, SR3, SR5, or SR10 (n = 5) to quantify TNF-α, IL-1β, IL-4, IL-6, IL-10, and BDNF levels (Mouse TNF-α DuoSet DY410, Mouse IL-1β/IL-1F2 DuoSet DY401, Mouse IL-4 DuoSet DY404, Mouse IL-6 DuoSet DY406, Mouse IL-10 DuoSet DY417, Human/Mouse BDNF DuoSet DY248, R&D Systems). Additionally, blood was collected and centrifuged at 4500 rpm for 10 min. Thereafter, plasma was separated, and IL-1β was quantified (Fig. S3). Following the manufacturer's instructions, cytokines and BDNF concentrations were determined using a capture enzyme-linked immunosorbent assay (ELISA). Samples were stored at -20 °C until they were used. Brain tissue was homogenized using 500 μL of lysis buffer solution containing a protease inhibitor cocktail. The mixture was then centrifuged at 15,000 rpm for 30 min at 4 °C, and the supernatant was collected. Samples were incubated on a capture antibody-coated plate for 24 h at 4 °C with PBS-Tween 20 (0.05%/0.5% BSA), washed three times, and incubated with a detection antibody for 2 h at room temperature. The attached antigen–antibody systems were detected using the TMB substrate. Optical density readings were done at 450 nm and 570 nm. The assay was performed in duplicate. Sample results were adjusted to pg/mg protein. Protein concentration was determined using the Bradford assay.

### Senescence-Associated Beta-Galactosidase (SA-β-gal) Activity Assay

The β-galactosidase activity was evaluated from control (CON) and ten-day sleep-restricted (SR10) group (n = 1) in 20 μm brain sections fixed with 4% paraformaldehyde as reported previously [19]. Slides were washed with PBS 1X and stained with a solution containing 20 mg/mL of X-gal (V394A, Promega, Madison, WI, United States) in dimethylformamide, 0.2 M citric acid/sodium phosphate buffer pH = 6, 100 mM potassium ferrocyanide, 5 M sodium chloride, and 1 M magnesium chloride. Slides were incubated in a wet chamber at 37 °C for ~ 12 h. Slides were photographed using 5 × and 20 × objectives, from an inverted microscope, Carl Zeiss VERT.A1. Subsequently, photomicrographs were analyzed using the Fiji free software [20].

### Immunofluorescence

Representative photomicrographs were obtained from Control (CON) and ten-day sleep-restricted (SR10) mouse (n = 1). Animals were anesthetized with an overdose of sodium pentobarbital i.p. When the animals were deeply anesthetized, 3 min of intracardial perfusion with 0.9% saline solution was performed, followed by 3 min of 4% PFA in PBS 1X. Brains were collected and post-fixed in 4% ice-cold PFA for 48 h. Brains were cryoprotected with 30% sucrose and 0.05% sodium azide in PBS and stored at 4 °C.

Brains were frozen using 2-methylbutane in a dry ice chamber and stored at − 40 °C until use. Serial coronal 20 μm-thick slices were obtained using a Leica CM1860 UV cryostat. Slices containing the bilateral dorsal hippocampus were collected in gelatin-coated slides and stored at 4 °C until immunofluorescence (IF) processing. Slides were washed with PBS 1X, followed by antigen retrieval (80–90 °C citrate buffer 1X, SIGMA C9999), permeabilization (PBS 1X containing 0.25% Triton X-100), and blocking (1% BSA in PBS 1X + 0.1% Tween 20). The slides were co-incubated 48 h at room temperature with the following primary antibodies (online resource 1): GFAP (Invitrogen, 14-9892-82, 1:1000), C3 complement system (GeneTex, GTX72994, 1:200) and Iba-1 (Abcam ab178847, 1:1000). Sections were washed with PBS 1X and co-incubated for two h with fluorescent secondary antibodies, Goat anti-mouse Alexa Fluor-594 (Jackson ImmunoResearch, 115-585-003, 1:500), Donkey anti-goat Alexa-Fluor 488 (Jackson ImmunoResearch, 705-545-147, 1:500), and Donkey Anti-Rabbit Alexa Fluor-680 (Jackson ImmunoResearch, 711-625-152, 1:500) and then washed with PBS 1X, incubated for 15 min with DAPI solution (Invitrogen, 4′,6-diamidino-2-phenylindole, dihydrochloride, D1306) and covered with anti-fade fluorescence mounting medium (Abcam, ab104135). Representative photomicrographs from CA1 and CA3 hippocampus, as well as the cerebral somatosensorial cortex, were obtained using a ZEISS LSM 880 confocal microscope equipped with a 20 × objective and Fiji free software.

### Statistical Analysis

All experiments and animal tests were conducted and evaluated by experimenters blinded to the different groups. Animals were allocated to each group with an equal probability of being selected through randomization. All met the age criteria (8–10 weeks old). The sample size was determined by the “resource equation method” [21]. To determine whether the data distribution complied with the normality assumption, the data were evaluated using the Shapiro–Wilk test. To assess the conformity of the numerical variables to a normal distribution, differences between groups in the datasets were analyzed by one‐way analysis of variance (ANOVA) tests. Dunnett’s test was used as a post hoc test to compare differences between pairs of groups. A p-value of less than 0.05 was considered statistically significant. Statistical analyses were performed using GraphPad Prism (Version 8.0.1). Data are presented as a mean ± standard deviation.

## Results

### Sleep Restriction-Induced Progressive Blood–Brain Barrier Dysfunction

**Fig. 1:**
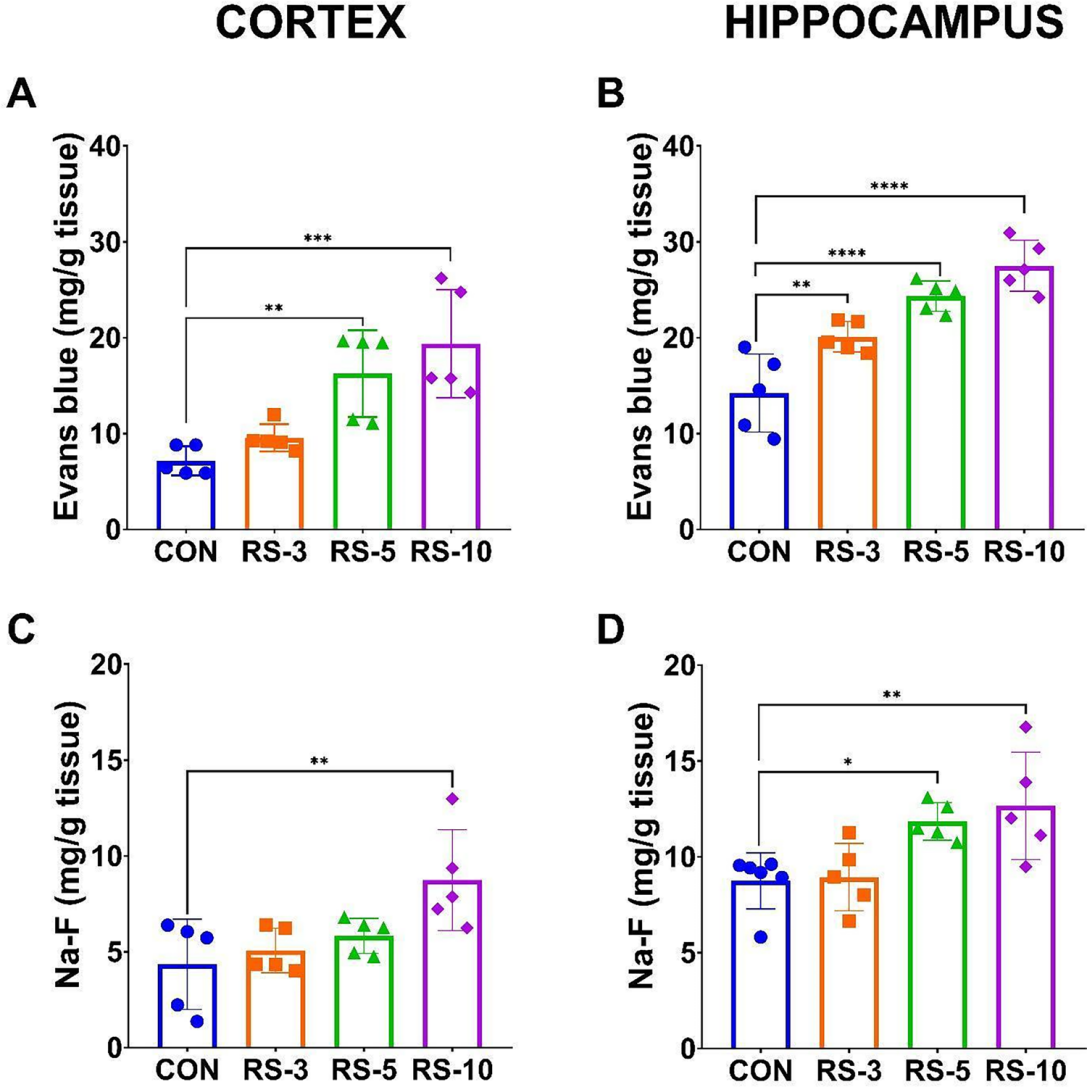
Sleep restriction disrupted the blood–brain barrier by increasing barrier permeability. Graphs show the increased BBB permeability in sleep-restricted mice for 3 (SR3), 5 (SR5), and 10 (SR10) days compared to intact controls (CON). Evans blue (**A** and **B**) and fluorescein-sodium (Na-F; **C** and **D**) evaluation in the cerebral cortex (**A** and **C**) and hippocampus (**B** and **D**). One-way ANOVA and a post hoc Dunnett’s test were performed. Mean ± standard deviation, n = 5 per group (duplicated), *p < 0.05, **p < 0.01; ***p < 0.001 compared to the CON

Control animals exhibited low BBB permeability to Evans blue and Na-F tracers. Sleep restriction promoted progressive BBB dysfunction, as shown by increased permeability to Evans blue in the cerebral cortex (F_3, 15_ = 11.2, *p* = 0.0004, Fig. 1A) and hippocampus (F_3, 16_ = 23.06, *p* = 0.0001, Fig. 1B) during the sleep restriction period. As shown in Fig. 1, BBB permeability for Evans Blue in the hippocampus increased as the sleep loss time elapsed, rising from day three of sleep restriction and reaching its maximum level at 10 days of sleep restriction (Fig. 1B). While in the cerebral cortex, there was a significant increase in permeability to Evans blue beginning at the fifth day of sleep restriction and reaching its maximum at 10 days of sleep loss (Fig. 1A). For Na-F, increased BBB permeability was observed after ten days of sleep restriction in the cerebral cortex (F_3, 16_ = 5.039, *p* = 0.0120, Fig. 1C) and after five days of sleep restriction in the hippocampus (F_3, 16_ = 5.642, *p* = 0.0078, Fig. 1D).

**Fig. 2:**
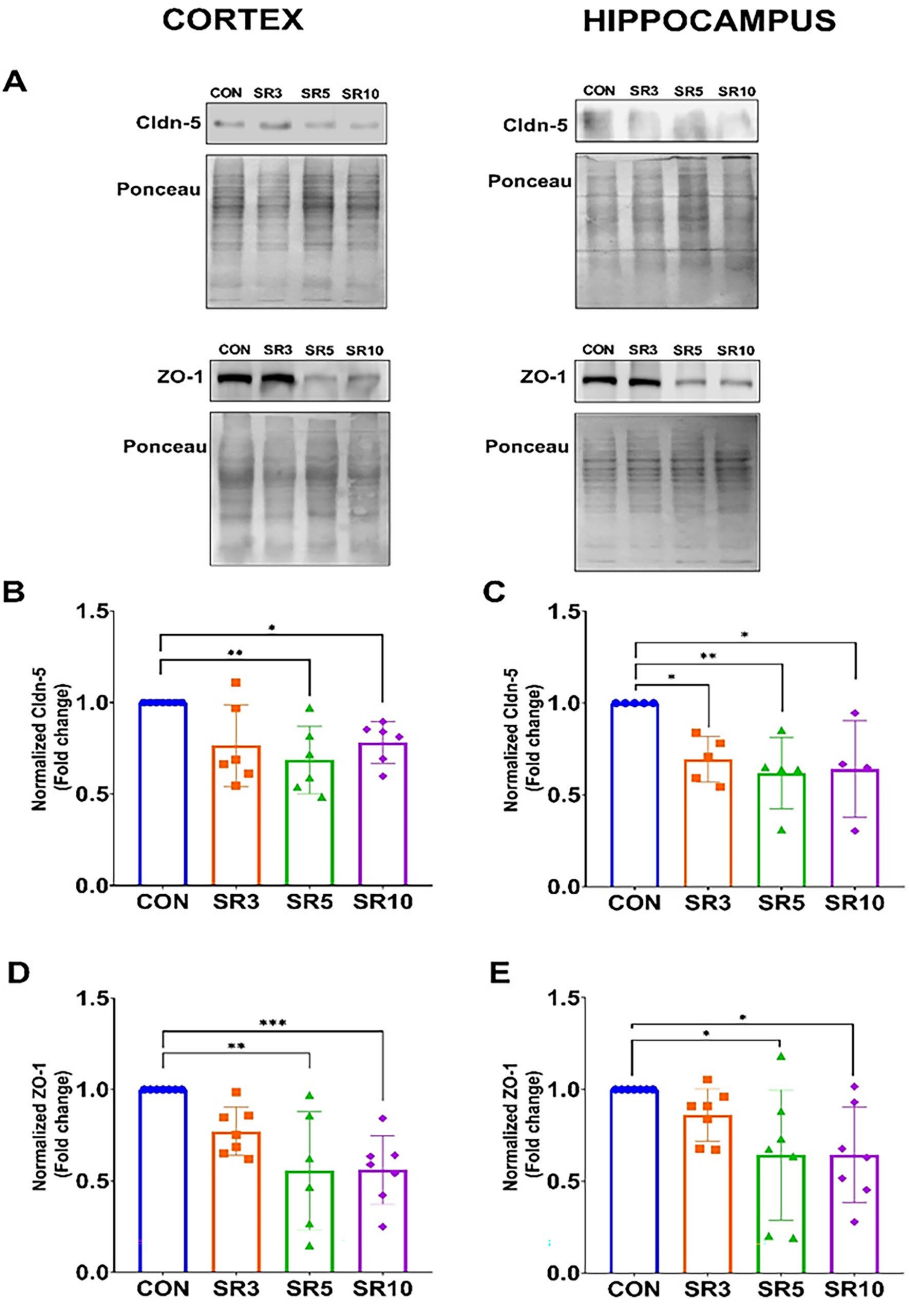
Sleep restriction disrupted the blood–brain barrier structure. Semi-quantitative analysis of tight junction protein expression in sleep-restricted mice at 3 (SR3), 5 (SR5), and 10 (SR10) days compared to intact controls (CON). Each panel displays a representative Western blot assay for each group (**A**). Graphs show claudin-5 (Cldn-5; **B** and **C**) and zonula occludens-1 (ZO-1; **D** and **E**) expression in the cerebral cortex and hippocampus. One-way ANOVA + and a post hoc Dunnett’s test were performed. Mean ± standard deviation, n = 5–7 per group (duplicated), *p < 0.05, **p < 0.01; ***p < 0.001 compared to the CON

Concurrently, decreased tight junction protein expression was observed during the sleep loss period (Fig. 2). Cldn-5 protein expression decreased in both regions: cerebral cortex (F_3, 20_ = 5.363, *p* = 0.0071, Fig. 2A, B) at five days of sleep restriction and in the hippocampus (F_3, 19_ = 5.883, *p* = 0.0051, Fig. 2A, C) from the third day of sleep loss, suggesting that in both regions the increased permeability is maintained after 10 days. The second tight junction protein evaluated was zonula occludens (ZO-1), which also decreased from the fifth day of sleep restriction in both structures, the cerebral cortex (F_3, 23_ = 8.309, *p* = 0.0006, Fig. 2A, D) and the hippocampus (F_4, 24_ = 4.013, *p* = 0.0190, Fig. 2E).

### Sleep Restriction Promoted Astroglial Reactivity

**Fig. 3:**
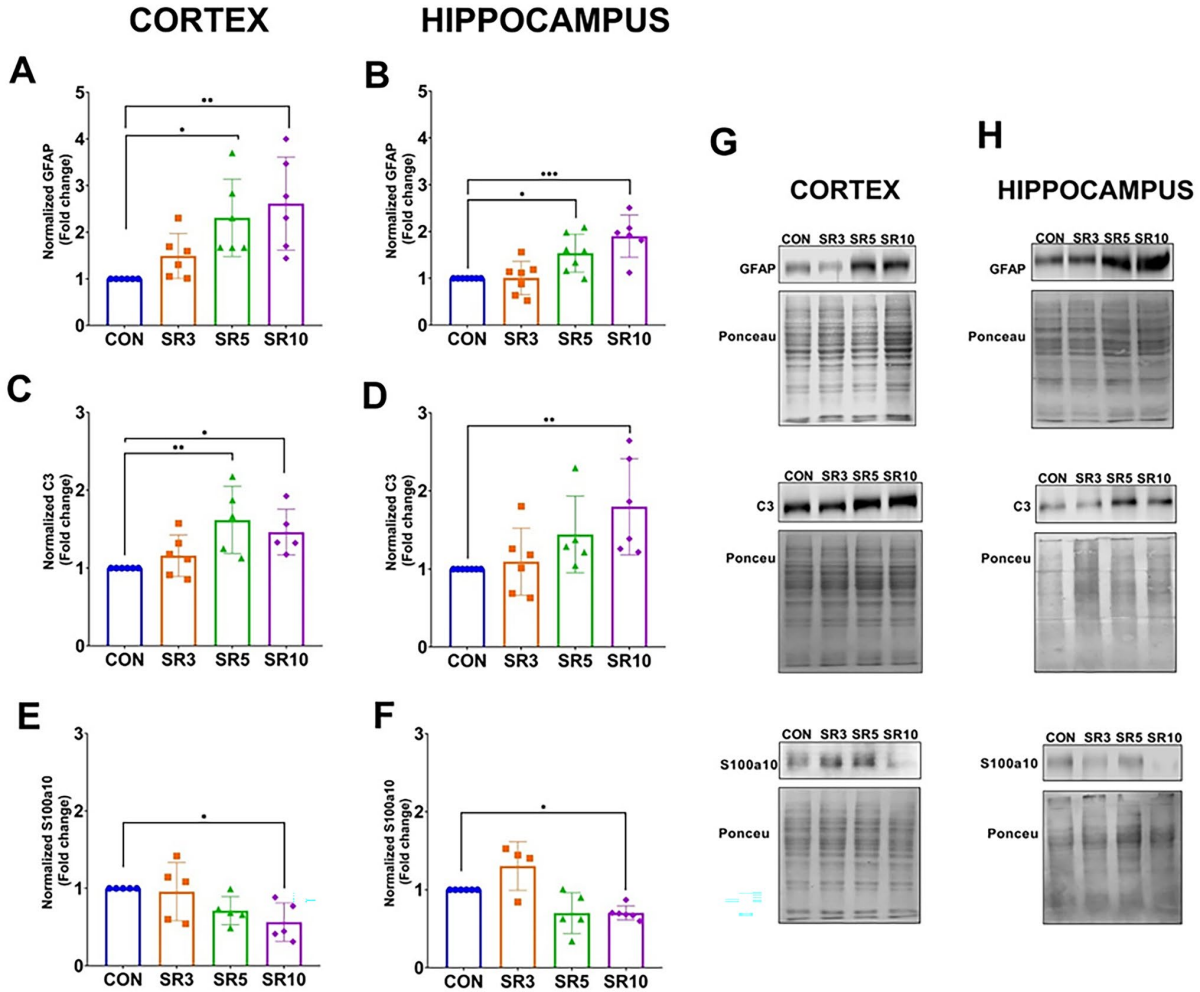
Astrocyte reactivity occurs after chronic sleep loss. Graphs illustrate the semi-quantitative analysis of GFAP, C3, and S100a10 protein expression in sleep-restricted mice at 3 (SR3), 5 (SR5), and 10 (SR10) days compared to intact controls (CON). GFAP (**A** and **B**), C3 protein (**C** and **D**), and S100a10 (**E** and **F**) expression in the cerebral cortex and hippocampus. Representative blots are shown for the cerebral cortex (**G**) and hippocampus (**H**). One-way ANOVA + and a post hoc Dunnett’s test were performed. Mean ± standard deviation, n = 4–7 per group (duplicated), *p < 0.05, **p < 0.01, ***p < 0.001 compared to the CON

Reactive astrogliosis has been described as occurring concurrently with BBB dysfunction after sleep loss [3]. However, the behavior of astrocytes during sleep restriction is unknown. Hence, GFAP was evaluated as an astroglial marker. GFAP levels were low in the cerebral cortex and hippocampus of the control animals that slept ad libitum (Fig. 3 A, B). In the sleep-restricted mice, GFAP levels increased at 5 days of sleep loss in the cerebral cortex (F_3, 20_ = 6.867, *p* = 0.0023, Fig. 3A, G), as well as in the hippocampus (F_3, 23_ = 10.40, *p* = 0.0002, Fig. 3B, H). Since reactive astroglia may induce neuroinflammation or present homeostatic functions, the primary markers of each response were evaluated. Neuroinflammatory reactive astroglia is associated with a neurotoxic response and can be identified by C3 protein expression, while the S100a10 protein expression is associated with an astroglial neuroprotective response [22]. Our data showed that C3 increased after 5 days of sleep restriction in the cerebral cortex (F_3, 18_ = 5.377, *p* = 0.0081, Fig. 3C, G), but in the hippocampus, higher levels were found at 10 days of sleep loss (F_3, 22_ = 5.969, *p* = 0.0039, Fig. 3D, H). On the contrary, S100a10 showed reduced levels after 10 days of sleep restriction in the cerebral cortex (F_3, 16_ = 3.629, p = 0.0360, Fig. 3E, G) and the hippocampus (F_3, 22_ = 8.055,* p* = 0.0008, Fig. 3F, H).

**Fig. 4:**
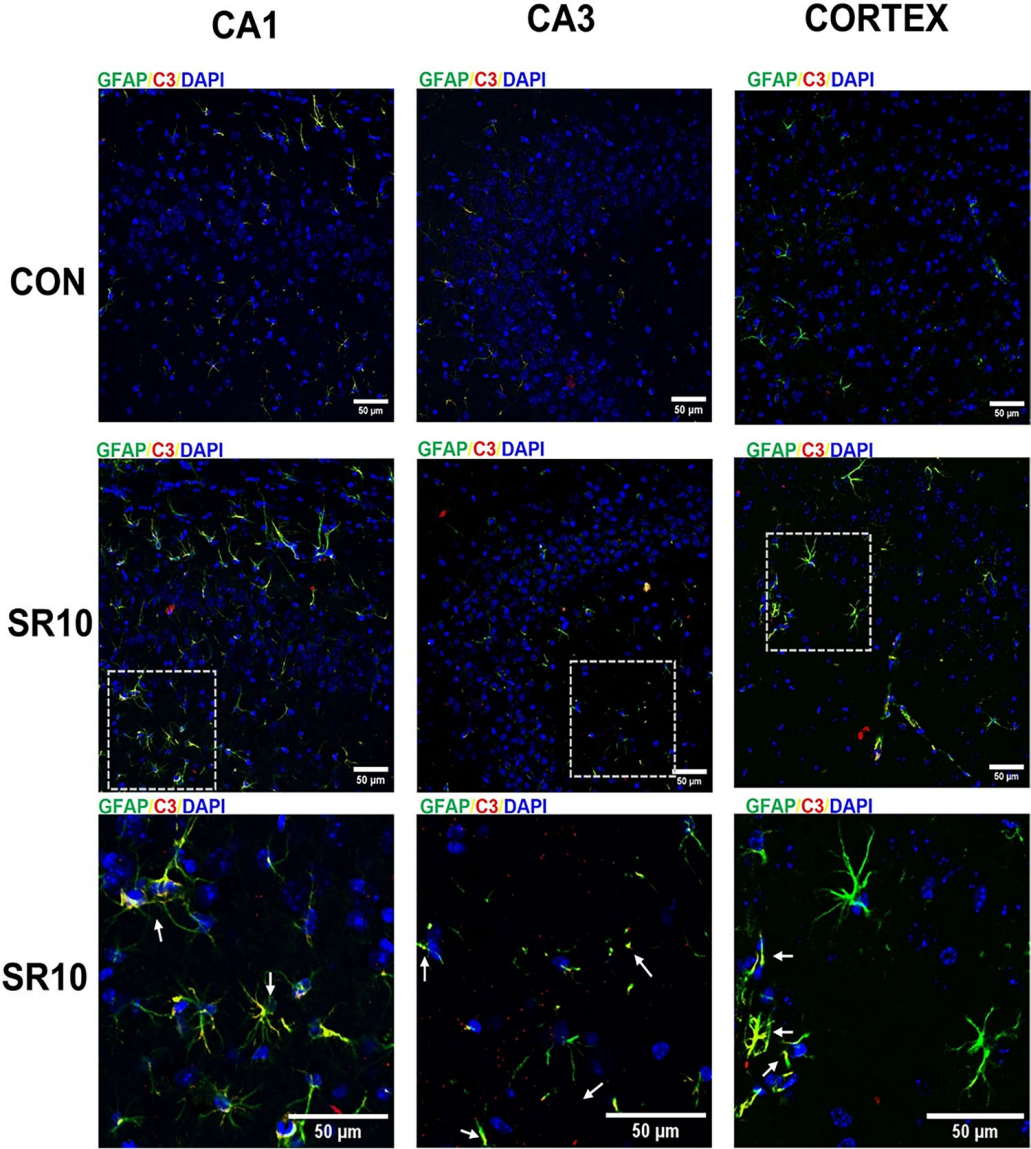
C3 complement protein is located in the astrocytes. Representative images of GFAP (green), C3 (red), and DAPI (blue) expression in the hippocampal regions CA1, CA3, and the cerebral cortex from control (CON) or 10-day sleep-restricted (SR10) mice are shown. The boxed area in SR10 is magnified in the bottom microphotographs; the arrows indicate C3^+^GFAP^+^ regions

**Fig. 5:**
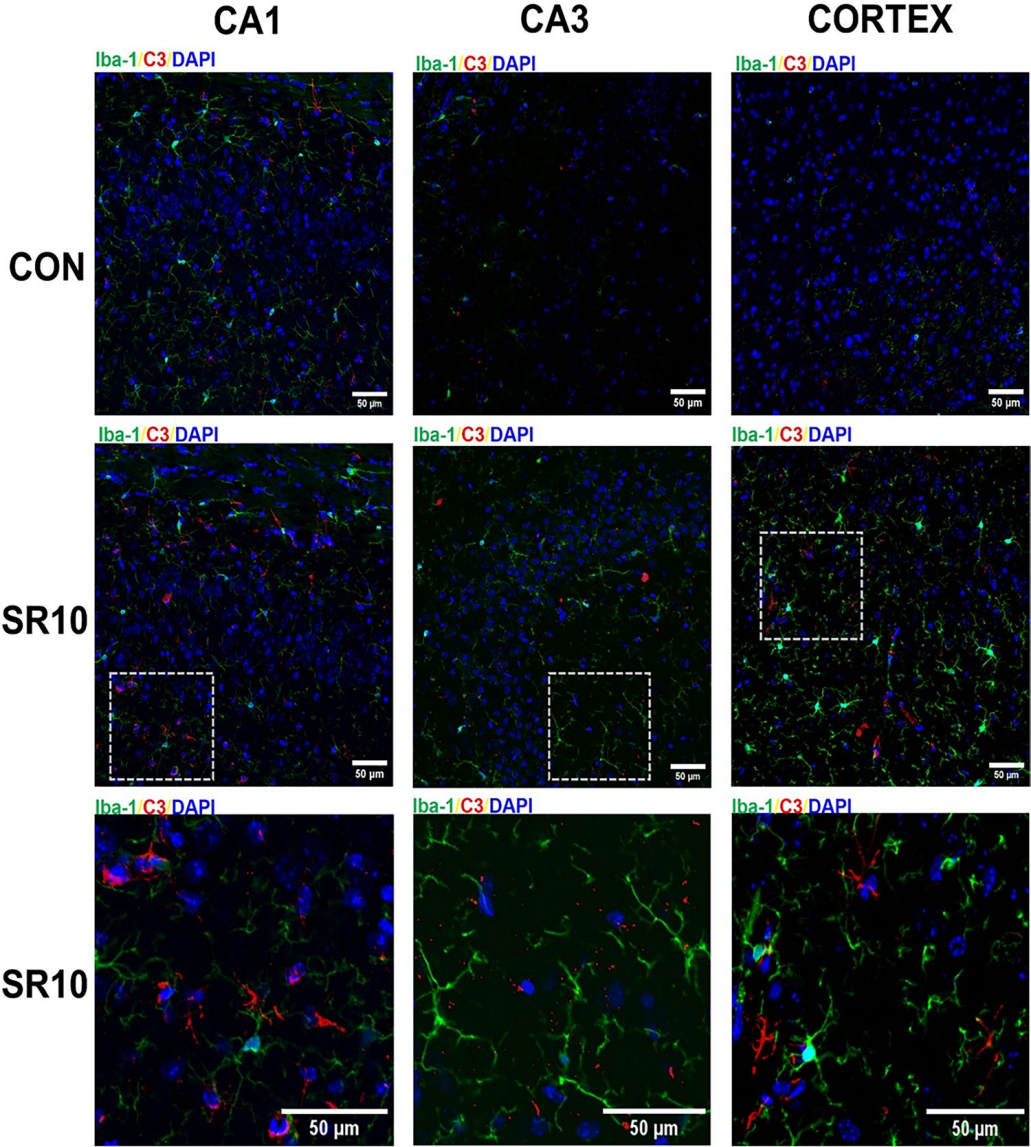
C3 complement protein is not colocalized with microglia in the chronic sleep restriction model. Representative images of Iba-1 (green), C3 (red), and DAPI (blue) expression in the hippocampal regions CA1, CA3, and the cerebral cortex from control (CON) or 10-day sleep-restricted (SR10) mice are shown. The boxed area in SR10 is magnified in the bottom microphotographs

To corroborate whether C3 is present in astrocytes, we evaluated the presence of C3 and GFAP in the hippocampus (CA1 and CA3 regions) and the cerebral cortex using confocal microscopy (Fig. 4; Fig. S1). In the representative photomicrograph, co-labeling of C3 and GFAP is observed, primarily in the hippocampus CA1 region (Fig. 4), suggesting that astrocytes may contribute to the increase of C3. We also evaluated the expression of C3 and Iba-1, a microglial marker; it appears that C3 is expressed in nearly all microglial processes (Fig. 5; Fig. S2).

### Sleep Loss Differentially Modified Cytokine Expression

**Fig. 6:**
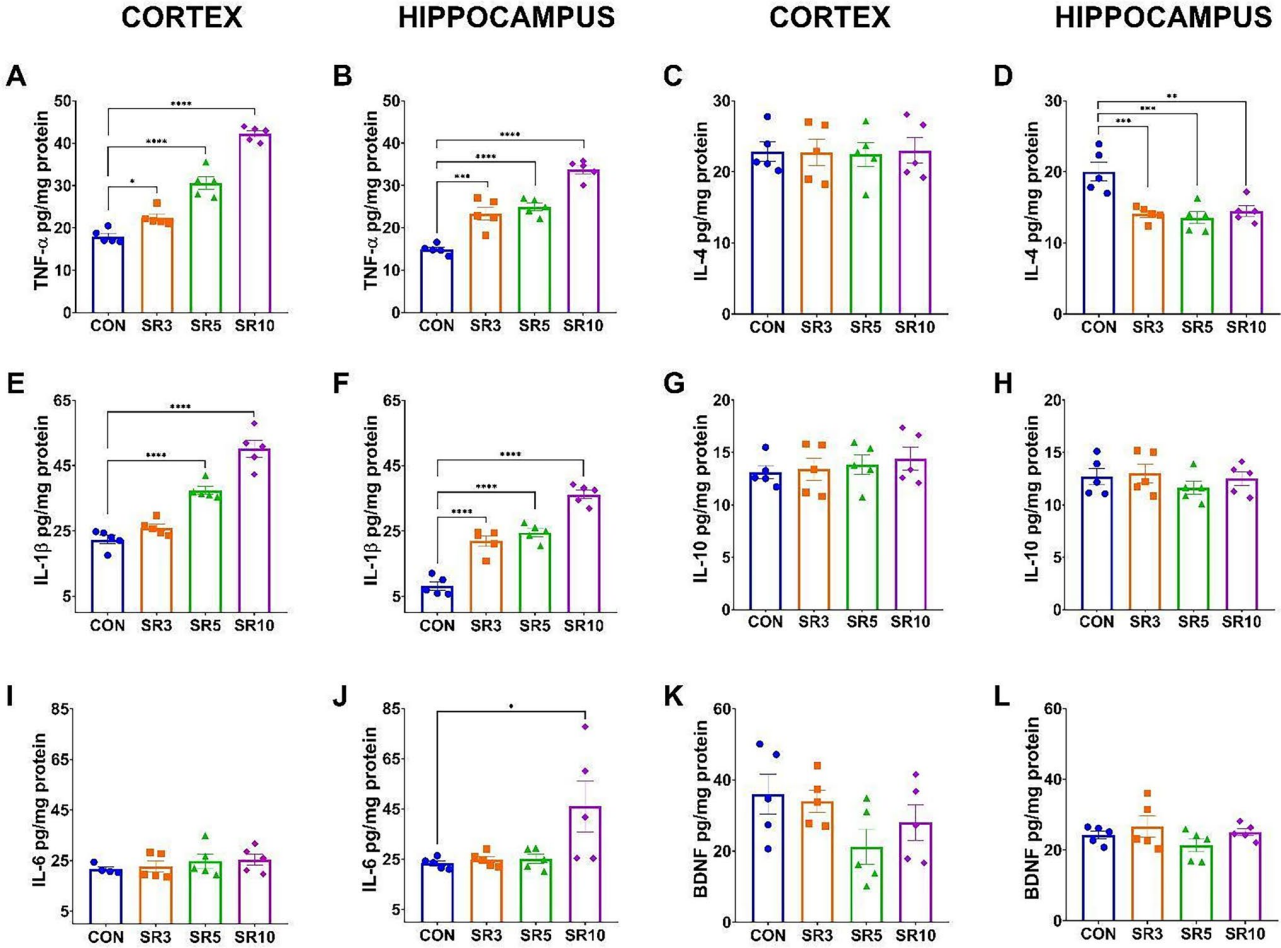
Sleep restriction induces neuroinflammation. Graphs show the levels of pro-inflammatory cytokines TNF-α (**A**, **B**), IL-1β (**C**, **D**), IL-6 (**E**, **F**), anti-inflammatory cytokines IL-4 (**G**, **H**), IL-10 (**I**, **J**), and BDNF (**K**, **L**) from the cerebral cortex (**A**, **C**, **E**, **G**, **I**, and **K**) and hippocampus (**B**, **D**, **F**, **H**, **J**, and **L**) of 3 (SR3), 5 (SR5), and 10 (SR10) days sleep-restricted mice, compared to intact controls (CON). Samples were quantified by ELISA. The assay was performed in duplicate, with n = 5 per group. One-way ANOVA + post hoc Dunnett’s test were performed. Mean ± standard deviation, *p < 0.05, **p < 0.01, ***p < 0.001 compared to the CON

Several cytokine levels differ in expression and production during aging and inflammatory events. We found increased levels of TNF-α from 3 days of sleep restriction, which raised while the sleep restriction days progressed in the cerebral cortex (F_3, 16_ = 111, *p* < 0.0001, Fig. 6A) and the hippocampus (F_3, 16_ = 52.70, *p* < 0.0001, Fig. 6B). IL-1β was higher after 5 and 3 days of sleep restriction in the cerebral cortex (F_3, 16_ = 57.78, *p* < 0.0001, Fig. 6E) and the hippocampus (F_3, 16_ = 70.78, *p* < 0.0001, Fig. 6F). Additionally, systemic IL-1β increased only at 10 days of sleep restriction (F_3, 16_ = 5.19, *p* < 0.0108, Fig. S3), suggesting that sleep restriction promotes pro-inflammatory cytokine production in the CNS before it increases in the periphery. No change was found in IL-4 levels in the cerebral cortex (Fig. 6C). Still, reduced levels were found in the hippocampus (F_3, 16_ = 11.18, *p* = 0.0003, Fig. 6D) from 3 days of sleep restriction, which is maintained at 10 days of sleep restriction (Fig. 6D). No change was found in IL-6 levels from cerebral cortex (Fig. 6I), while in the hippocampus shown an increase in comparison to the control group after 10 days of sleep restriction (F_3, 16_ = 4.277, *p* = 0.0213, Fig. 6J). IL-10 and BDNF levels were not modified by sleep restriction in either structure (Fig. 6G, H and K, L). These results demonstrate a differential expression in the inflammatory profile depending on the brain region.

### Chronic Sleep Loss Promoted Cellular Senescence in the Central Nervous System

**Fig. 7:**
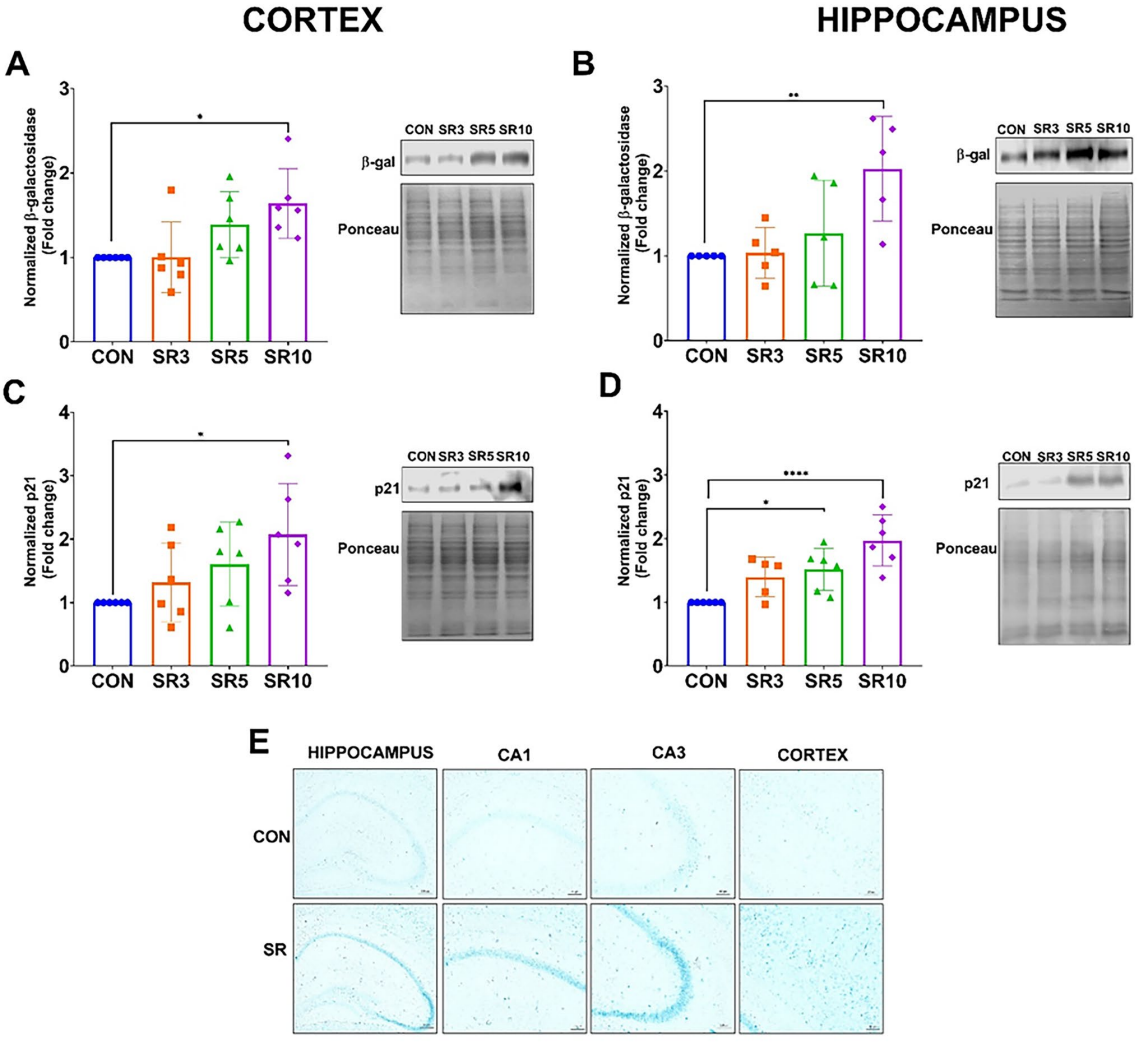
Chronic sleep loss induces cellular senescence in the central nervous system. Chronic sleep loss increased the expression of the senescence marker β-galactosidase and p21 in sleep-restricted mice at 3 (SR3), 5 (SR5), and 10 (SR10) days as compared to intact controls (CON). β-galactosidase (**A** and **B**) and p21 (**C** and **D**) expression in the cerebral cortex (**A** and **C**) and hippocampus (**B** and **D**). The β-galactosidase activity representative assay is shown in (**E**). One-way ANOVA + and a post hoc Dunnett’s test were performed. Mean ± standard deviation, n = 5–6 per group (in duplicates), *p < 0.05, ***p < 0.001 compared to the CON

Senescence can be induced by oxidative stress and an inflammatory environment [15]. Since sleep deprivation promotes neuroinflammation and oxidative stress [10, 23], two senescence markers (β-galactosidase and p21) were determined in the cerebral cortex and hippocampus of control and sleep-restricted mice. Our data showed increased levels of β-galactosidase after 10 days of sleep restriction in the cerebral cortex (F_3, 20_ = 4.798, *p* = 0.0112, Fig. 7A) and in the hippocampus (F_3, 16_ = 5.292, *p* = 0.0100, Fig. 7B). The amount of p21 is increased in the SR10 group in both evaluated regions: the cerebral cortex (F_3, 20_ = 3.398, *p* = 0.0379, Fig. 7C) and the hippocampus (F_3,19_ = 10.48, *p* = 0.0003, Fig. 7D). The increase was observed from 5 days of sleep loss. Additionally, SA-β-gal activity assays confirmed the higher levels of β-galactosidase in the cerebral cortex, CA1, and CA3 regions of the hippocampus from the SR10 group compared to the CON group (Fig. 7E, F).

## Discussion

Sleep disturbances represent a significant and growing public health issue that affects all age groups [24]. In Mexico, 37% of the adult population presented sleep difficulties [25]. However, this problem became apparent during the COVID-19 pandemic, when up to 40% of the general population reported poor sleep quality [26]. Sleep is essential for regulating multiple physiological processes in organisms, including mental health, memory, cognition, as well as immune regulation and maintenance of brain homeostasis [8, 27–29]. Despite this, the mechanistic consequences of sleep loss on brain physiology remain incompletely elucidated.

In this study, we provide evidence that chronic sleep restriction in young adult C57BL/6 mice induces a progressive and region-specific disruption of BBB function, accompanied by neuroinflammation and cellular senescence in both the cerebral cortex and hippocampus. BBB permeability increased since the early days of sleep restriction, followed by increased GFAP and C3 expression, depicting astroglial reactivity (Figs. 1, 2, 3). Evans blue permeability was altered as early as the fifth day of sleep restriction in the cerebral cortex and on day 3 in the hippocampus (Fig. 1A, B). However, an increased Na-F tracer occurred after 5 days in the cortex and 10 days of sleep restriction in the hippocampus (Fig. 1C, D). Our results also showed that the BBB was more permeable to Evans blue than Na-F. Evans blue is a tracer of 961 Da that, upon administration, binds to serum albumin, acquiring a molecular weight of 69 kDa [30], therefore, it is used as an indicator of albumin entry to the brain parenchyma. Despite its size, the increase in BBB permeability to albumin may occur earlier than that of other molecules, even of lower molecular weight [31]. However, some studies have indicated that Na-F can enter first [30].

The increased BBB permeability is known to exacerbate neuroinflammation in chronic sleep loss [2]. Conversely, Cldn-5 is the major tight junction protein that impedes paracellular diffusion of blood-borne solutes. Our research group previously reported that 10 days of sleep restriction decreased the expression of tight junction proteins in the rat brain, including cldn-5, ZO-1, and occludin [11]. Here, we observed that after five days of sleep restriction, there is an increase in BBB permeability to Na-F (Fig. 1C, D), which is concomitant to the decreased expression levels of cldn-5 and ZO-1 (Fig. 2). Considering that Na-F is a negatively charged hydrophilic molecule, in addition to the fact that its main entry into the brain parenchyma is through the paracellular pathway [30], a reduction in tight junction protein expression is necessary to allow its access until 10 days of sleep restriction, as we show here and was previously reported [10].

Astrocytes are critical regulators of the BBB in physiological conditions [32, 33] due to their release of various trophic factors that promote the expression of tight junction proteins, such as cldn-5 [34, 35]. However, the increase in reactive astrocytes has been described during inflammatory conditions, mainly in acute or high-grade inflammatory events [22, 36, 37]. Reactive astrocytes exhibit elevated levels of GFAP and undergo cellular morphological changes. Although sleep restriction is associated with a low-grade inflammation [2, 38, 39], we observed increased GFAP protein expression after the fifth day of sleep restriction (Fig. 3A, B) in both evaluated regions (complete tissue), concomitant with the increase in the C3 complement component at 5 days of sleep restriction in the cerebral cortex and 10 days of sleep restriction in the hippocampus (Fig. 3C, D). In contrast, the expression of the S100a10 decreased after 10 days of sleep restriction in both the cerebral cortex and hippocampus (Fig. 3E–H).

Modulation of the reactive astroglial profiles has been observed in different neuroinflammatory conditions [36, 40], including LPS-induced neuroinflammation [22] and chronic post-surgical pain [36]. An increase in the expression of both GFAP and the C3 protein was observed after the 4th day post-surgery, with its maximum peaks on days 7 and 14,a decrease in S100a10 was also observed [36], which correlates with our data. Moreover, we found that C3 was expressed in GFAP + cells but not in Iba-1 + cells (Figs. 4 and 5), as previously reported [41]. C3 seems expressed near microglial processes (Fig. 5). This finding may be related to the fact that C3 receptors (C3aR) are located in various cells in the CNS, including microglia, astrocytes, neurons, and endothelial cells [9, 42]. However, our group has reported that Iba-1 expression is higher after 10 days of sleep loss [3]. Additionally, Xie and colleagues found that after 2 months of sleep fragmentation, the Iba-1 marker was increased compared to the control group [43].

Under physiological conditions, C3 is involved in synaptic pruning [8, 44]. Nevertheless, in neuroinflammation, reactive astrocytes increase C3 expression, which is linked to endothelial cell activation. This effect has been reported in both in vitro models and in human brain tissue [41]. Furthermore, aged animals have shown that C3 promotes BBB dysfunctions via the C3aR located in endothelial cells, leading to a reduction in TJ proteins, in addition to an increase in adhesion molecules (VCAM) and damage to the blood vessels' morphology [9]. This could be one of the mechanisms by which astrocytes promote BBB dysfunction through the C3/C3aR pathway, leading to increased BBB permeability and structural damage (Figs. 1 and 2).

Concurrently with the C3 protein and GFAP, the levels of the proinflammatory cytokine TNF-α increase during neuroinflammation [22, 45]. TNF-α has been considered the primary inducer of neurotoxic astrocytes. Additionally, microglia may be responsible for this induction through the secretion of TNF-α, C1q, and IL-1α, which can induce reactivity via CXCR7, subsequently activating the PI3K/AKT pathway [22, 36]. We found that 3 days of chronic sleep restriction increased the levels of pro-inflammatory cytokine TNF-α in both evaluated regions (Fig. 6A, B). However, the increase in GFAP and C3 is observed from day 5 of sleep restriction (Fig. 3), suggesting that microglia may induce astroglial reactivity. Wadhwa and colleagues [46] reported that 48 h of sleep deprivation promoted an increase in the microglial marker Iba-1 in the hippocampus, but not GFAP. Additionally, the C3 complement component and TNF-α, IL-1β, and IL-6 were elevated. At the same time, IL-4 decreased, suggesting that microglia may be activated very early during acute periods of sleep loss, leading to astrocyte activation after 5 days of chronic sleep restriction.

The early activation of microglia may play a fundamental role against other cerebral regions; the hippocampus has the highest microglial proliferative rate, due to the proximity to the proliferative zone [47], which can affect the density and the rapid response after a stimulus, such as sleep restriction, contributing to maintain inflammatory environment as well as neurotoxic astrocytes. In addition, a previous report showed that the hippocampus exhibits BBB hyperpermeability even after sleep recovery periods, allowing for the improvement of BBB function in other brain regions [17], suggesting that this region responds differently.

Interestingly, the hippocampus appeared to be more susceptible to damage with raised IL-1β levels after 3 days of sleep restriction, whereas the cerebral cortex presented an increase after 5 days of sleep restriction. IL-6 increased after 10 days of sleep restriction (Fig. 6), while IL-4 decreased from 3 days of sleep restriction but only in the hippocampus (Fig. 6D). IL-6 is considered a secondary cytokine since TNF-α and IL-1β may induce it [48]. Here, we found that TNF-α and IL-1β levels increased in the first days of sleep restriction, primarily in the hippocampus, which also showed an increase in IL-6 after 10 days of sleep restriction. Still, no changes were observed in the cerebral cortex (Fig. 6), as reported by Guisasola et al. [48].

Since sleep restriction induces neuroinflammation [10], we evaluated the pro-inflammatory cytokines TNF-α, IL-1β, and IL-6 (Fig. 6), which have also been described as part of the SASP and the secretory profile of reactive astrocytes [15]. Consequently, distinguishing whether the outcomes observed at day 10 of sleep restriction are predominantly driven by neuroinflammation, senescence, or their interaction remains a complex task.

To reliably identify cellular senescence, current guidelines recommend assessing at least three distinct senescence markers [16]. In this study, we evaluated three SASP-associated cytokines (TNF-α, IL-1β, and IL-6), the cell cycle inhibitor p21, as well as β-galactosidase expression and activity, which serve as markers of increased lysosomal content. We found that the pro-inflamatory cytokines TNF-α, IL-1β (Fig. 6) increase from the third day of sleep restriction, but after 10 days, the increase is exacerbated, which corresponds to increased levels of IL-6 (Fig. 6), β -gal and p21 (Fig. 7). Therefore, the local inflammatory environment observed during sleep loss may also be linked to increased cellular senescence in the CNS.

BBB dysfunction may be induced by a neuroinflammatory environment, as presented in this study. Our results showed that early BBB dysfunction, accompanied by reactive neuroinflammatory astrocytes, appeared to promote cellular senescence in the CNS. Currently, no reports have demonstrated that sleep restriction increases the markers of cellular senescence in the CNS in young adult models. In our chronic sleep restriction model, 8-to 10-week-old mice exhibited increased cellular senescence in the hippocampus and cerebral cortex, as indicated by the increase in senescence markers after 10 days of sleep restriction in both evaluated regions (Fig. 7).

Our findings showed that the hippocampus is more susceptible to cellular damage under sleep loss conditions than the cerebral cortex since we found from 3 days of chronic sleep restriction BBB hyperpermeability to Evans blue (Fig. 1B), decreased levels of Cldn-5 (Fig. 2) and IL-4 opposite to the increment in the TNF-α, IL-1β (Fig. 6); p21 and IL-6 increase after 5 and 10 days of sleep restriction, respectively.

Senescent cells can be generated by various factors, including telomere shortening and exposure to stressors [49] such as DNA damage agents, reactive oxygen species (ROS) [49], and even a pro-inflammatory environment [50], which are increased during sleep loss [51–53].

The increase in ROS may be associated with astroglial reactivity as astrocytes are one of the primary producers of glutathione [54]. Reactive astrocytes may decrease glutathione production under sleep loss conditions, thereby promoting the induction of senescence in the CNS. The senescence markers observed in our study have been reported to increase in the senescent astroglia [15, 55]. Strikingly, poor sleep quality has been implicated in both the earlier onset and exacerbation of Alzheimer’s disease [56]. It is plausible that the premature induction of cellular senescence, a consequence of inadequate sleep, may serve as a mechanistic link in this association. In the present study, we assessed senescence markers in lysates from the cerebral cortex and hippocampus. However, the specific cell types contributing to the observed senescent phenotype remain to be elucidated.

## Conclusion

In summary, our findings demonstrate that sleep loss induces progressive blood–brain barrier dysfunction in both the cerebral cortex and hippocampus of young mice, accompanied by pronounced local neuroinflammation, as evidenced by increased expression of GFAP, C3, and the pro-inflammatory cytokines TNF-α, IL-1β, and IL-6. Chronic sleep deprivation also led to decreased levels of IL-4 and the neuroprotective astroglial marker S100a10, suggesting a shift away from a supportive astroglial phenotype and a potential reduction in astroglial trophic support.

Critically, we provide evidence that chronic sleep loss promotes cellular senescence within the CNS, particularly in the hippocampus, as indicated by elevated levels of the senescence markers p21 and β-galactosidase after 10 days of sleep restriction.

Although research in this area is limited, our findings offer novel insights into the mechanisms by which chronic sleep deprivation may accelerate cellular senescence in the young brain. Further studies are essential to clarify whether chronic sleep restriction, even in the short term and at an early age, biologically impacts the generation of senescent cells and whether these effects will have short-term or long-term repercussions.

## Supplementary Information

Below is the link to the electronic supplementary material.

Supplementary file1 (PDF 186 KB)

Supplementary file2 (PDF 3038 KB)

Supplementary file3 (PDF 28421 KB)

## Data Availability

The datasets used and/or analyzed during the current study are available from the corresponding author upon reasonable request.

## Abbreviations

BBB: Blood–brain barrier
CCL2: Chemokine monocyte chemoattractant protein-1
Cldn-5: Claudin-5
CNS: Central nervous system
CON: Control group
ELISA: Enzyme-linked immunosorbent assay
GFAP: Glial fibrillary acid protein
Iba-1: Ionized calcium-binding adaptor molecule 1
IL-6: Interleukin-6
Il-8: Interleukin-8
Na-F: Fluorescein sodium salt
p21: Cyclin-dependent kinase inhibitor 1
ROS: Reactive oxygen species
SR: Sleep restriction
SASP: Senescence associated secretory phenotype
TEER: Transendothelial electrical resistance
ZO-1: Zonula occludens 1
β-gal: β-Galactosidase protein
