# Supplementary material for: Progressive Blood–Brain Barrier Disruption in Sleep-Restricted Young Mice: Cellular Senescence and Neuroinflammation Crosstalk
Source: Neurochem Res. 2025 Aug 18;50(5):269. doi: 10.1007/s11064-025-04510-y (PMC12361337; doi:10.1007/s11064-025-04510-y)
Supplement: Supplementary file 1 — Supplementary file1 (PDF 186 KB) [file 11064_2025_4510_MOESM1_ESM.pdf]

ARTICLE: BLOOD-BRAIN BARRIER DYSFUNCTION DURING SLEEP LOSS IS ASSOCIATED WITH CELLULAR SENESCENCE AND NEUROINFLAMMATION

JOURNAL: CELLULAR AND MOLECULAR NEUROBIOLOGY

AUTHORS: Jessica J. Avilez-Avilez, J. Enrique García-Aviles , Ricardo Jair Ramírez-Carreto, Verónica Salas-Venegas, Mara A. Guzmán-Ruiz, Ma. Fernanda Medina-Flores, Mina Königsberg, Anahí Chavarria*, Beatriz Gómez-González*.

CORRESPONDING AUTHORS:

Anahí Chavarría:

Unidad de Medicina Experimental "Dr. Ruy Pérez Tamayo", Facultad de Medicina, Unidad 502 Hospital General de México, Dr. Balmis 148, Col. Doctores, Cuauhtémoc,Mexico City, Mexico, 06726.

Telephone: +52 5556232682

e-mail: anahi.chavarria@facmed.unam.mx

Beatriz Gómez-González:

Address: Area of Neurosciences, Dept. Biology of Reproduction, CBS, Universidad Autónoma Metropolitana, Unidad Iztapalapa, Av. San Rafael Atlixco No. 186, Col. Leyes de Reforma 1ª Sección, Iztapalapa, Mexico City, Mexico, 09310.

Telephone: +52 55 5804 6559

e-mail: bgomezglez@gmail.com & bgomez@izt.uam.mx

TABLE 1. PRIMARY ANTIBODIES DETAILS

TECHNIQUE	ANTIBODY AND SOURCE	DILUTION	VALIDATION SOURCE
WESTERN BLOT	**Claudin-5** Biorbyt, cat# orb160461	1:500	(Medina-Flores et al., 2020)
	**ZO-1** Life Technologies cat# 40-2200, (by Thermo Fisher Scientific)	1:1000	(Richards et al., 2022) https://www.thermofisher.com/antibody/product/ZO-1-Antibody-Polyclonal/40-2200?imageId=4318
	**GFAP** Abcam, cat# ab4648	1:1000	(Hurtado-Alvarado et al., 2016)
	**C3** Abcam, cat# ab200999	1:1000	(Schartz et al., 2023)
	**S100a10** Novus Biologicals, cat# NBP1 89370	1:1000	(Vay et al., 2021) https://www.novusbio.com/products/s100a10-antibody_nbp1-89370#datasheet
	**β-galactosidase** Santa Cruz Biotechnology, cat# sc-65670	1:1000	(Borgonetti & Galeotti, 2022)
	**p21** Santa Cruz Biotechnology, cat# sc-6246	1:1000	(Siraj et al., 2024)
IMMUNOFLUORESCENCE	**GFAP** Invitrogen, cat# 14-9892-82 (by Thermo Fisher Scientific)	1:1000	(Mok et al., 2023)
	**C3** GeneTex, cat# GTX72994	1:200	(Kohn et al., 2021)
	**Iba-1** Abcam, cat# ab178847	1:1000	(Yu et al., 2018)

REFERENCES (INCLUDED IN THE MANUSCRIPT)

Borgonetti, V., & Galeotti, N. (2022). Rosmarinic Acid Reduces Microglia Senescence: A Novel Therapeutic Approach for the Management of Neuropathic Pain Symptoms. *Biomedicines*, *10*(7), Article 7. https://doi.org/10.3390/biomedicines10071468

Hurtado-Alvarado, G., Domínguez-Salazar, E., Velázquez-Moctezuma, J., & Gómez-González, B. (2016). A2A Adenosine Receptor Antagonism Reverts the Blood-Brain Barrier Dysfunction Induced by Sleep Restriction. *PLOS ONE*, *11*(11), e0167236. https://doi.org/10.1371/journal.pone.0167236

Kohn, M., Lanfermann, C., Laudeley, R., Glage, S., Rheinheimer, C., & Klos, A. (2021). Complement and Chlamydia psittaci: Non-Myeloid-Derived C3 Predominantly Induces Protective Adaptive Immune Responses in Mouse Lung Infection. *Frontiers in Immunology*, *12*. https://doi.org/10.3389/fimmu.2021.626627

Medina-Flores, F., Hurtado-Alvarado, G., Contis-Montes de Oca, A., López-Cervantes, S. P., Konigsberg, M., Deli, M. A., & Gómez-González, B. (2020). Sleep loss disrupts pericyte-brain endothelial cell interactions impairing blood-brain barrier function. *Brain, Behavior, and Immunity*. https://doi.org/10.1016/j.bbi.2020.05.077

Mok, K. K.-S., Yeung, S. H.-S., Cheng, G. W.-Y., Ma, I. W.-T., Lee, R. H.-S., Herrup, K., & Tse, K.-H. (2023). Apolipoprotein E ε4 disrupts oligodendrocyte differentiation by interfering with astrocyte-derived lipid transport. *Journal of Neurochemistry*, *165*(1), 55–75. https://doi.org/10.1111/jnc.15748

Richards, M., Nwadozi, E., Pal, S., Martinsson, P., Kaakinen, M., Gloger, M., Sjöberg, E., Koltowska, K., Betsholtz, C., Eklund, L., Nordling, S., & Claesson-Welsh, L. (2022). Claudin5 protects the peripheral endothelial barrier in an organ and vessel-type-specific manner. *eLife*, *11*, e78517. https://doi.org/10.7554/eLife.78517

Schartz, N. D., Aroor, A., Li, Y., Pinzón-Hoyos, N., & Brewster, A. L. (2023). Mice deficient in complement C3 are protected against recognition memory deficits and astrogliosis induced by status epilepticus. *Frontiers in Molecular Neuroscience*, *16*. https://doi.org/10.3389/fnmol.2023.1265944

Siraj, Y., Aprile, D., Alessio, N., Peluso, G., Di Bernardo, G., & Galderisi, U. (2024). IGFBP7 is a key component of the senescence-associated secretory phenotype (SASP) that induces senescence in healthy cells by modulating the insulin, IGF, and activin A pathways. *Cell Communication and Signaling*, *22*(1), 540. https://doi.org/10.1186/s12964-024-01921-2

Vay, S. U., Olschewski, D. N., Petereit, H., Lange, F., Nazarzadeh, N., Gross, E., Rabenstein, M., Blaschke, S. J., Fink, G. R., Schroeter, M., & Rueger, M. A. (2021). Osteopontin regulates proliferation, migration, and survival of astrocytes depending on their activation phenotype. *Journal of Neuroscience Research*, *99*(11), 2822–2843. https://doi.org/10.1002/jnr.24954

Yu, J., Li, X., Matei, N., McBride, D., Tang, J., Yan, M., & Zhang, J. H. (2018). Ezetimibe, a NPC1L1 inhibitor, attenuates neuronal apoptosis through AMPK dependent autophagy activation after MCAO in rats. *Experimental Neurology*, *307*, 12–23. https://doi.org/10.1016/j.expneurol.2018.05.022
