# Supplementary material for: Progressive Blood–Brain Barrier Disruption in Sleep-Restricted Young Mice: Cellular Senescence and Neuroinflammation Crosstalk
Source: Neurochem Res. 2025 Aug 18;50(5):269. doi: 10.1007/s11064-025-04510-y (PMC12361337; doi:10.1007/s11064-025-04510-y)

BLOOD-BRAIN BARRIER DYSFUNCTION DURING SLEEP LOSS IS ASSOCIATED WITH CELLULAR SENESCENCE AND NEUROINFLAMMATION

Jessica J. Avilez-Avilez[1,2], J. Enrique García-Aviles[1,3#], Ricardo Jair Ramírez-Carreto[4,5#], Verónica Salas-Venegas[4,6], Mara A. Guzmán-Ruiz[3], Ma. Fernanda Medina-Flores[7], Mina Königsberg[8], Anahí Chavarria[4*], Beatriz Gómez-González[2*].

GROUPS

- CON: CONTROL GROUP
- SR3: SLEEP RESTRICTED MICE DURING 3 DAYS
- SR5: SLEEP RESTRICTED MICE DURING 5 DAYS
- SR10: SLEEP RESTRICTED MICE DURING 10 DAYS

PROTEINS

- Claudin-5 (~23 kDa)
- Zónula ocludens:~200kDa
- Glial Fibrilar Acid Protein (GFAP):~50 kDa
- C3 complement component:~115 kDa
- S100a10:~10 kDa
- β-galactosidase: :~120 kDa
- p21: 21 kDa

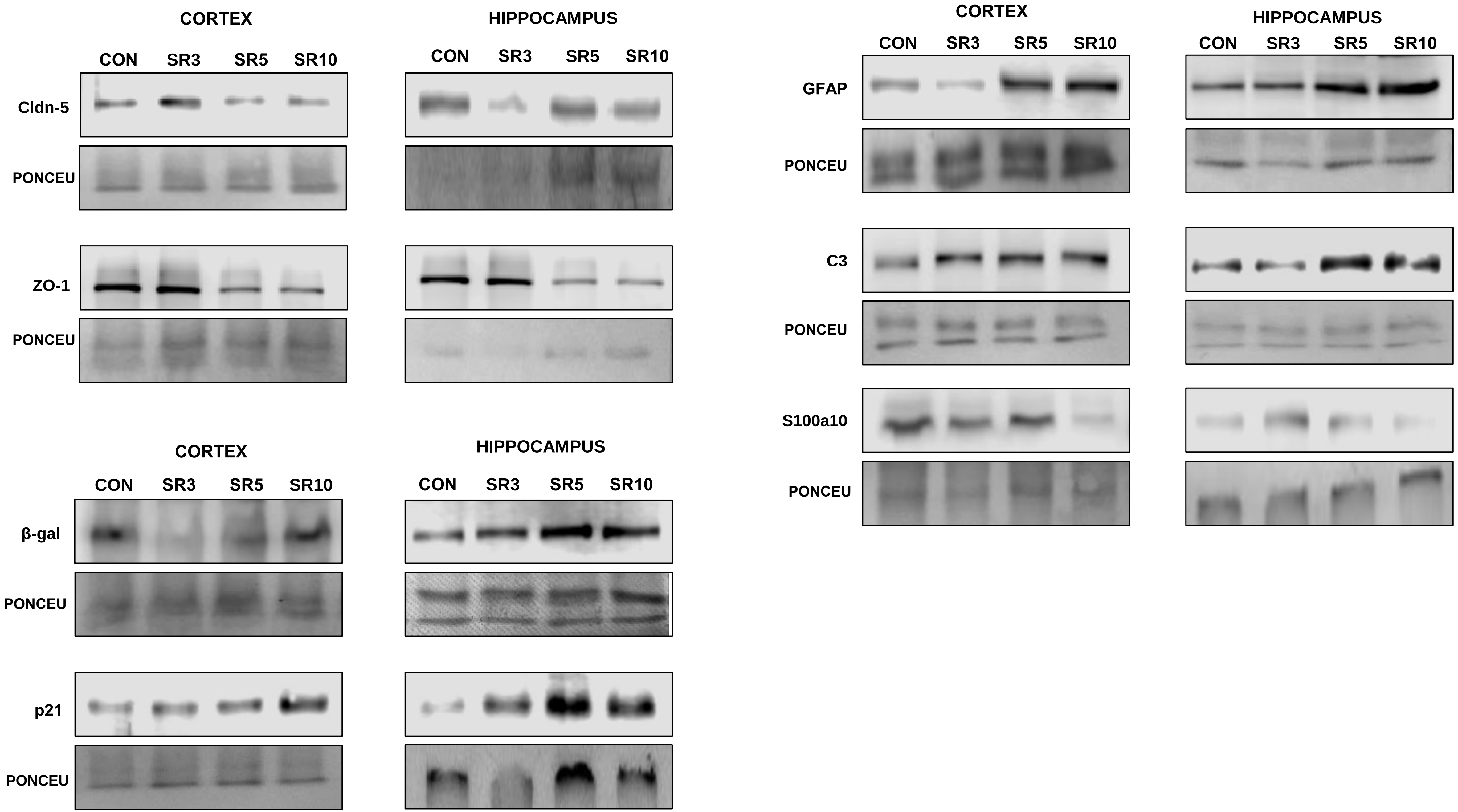
CORTEX
HIPPOCAMPUS
CON
SR3
SR5
SR10
Cldn-5
PONCEU
ZO-1
β-gal
p21
GFAP
C3
S100a10

CEREBRAL CORTEX: CLAUDIN-5

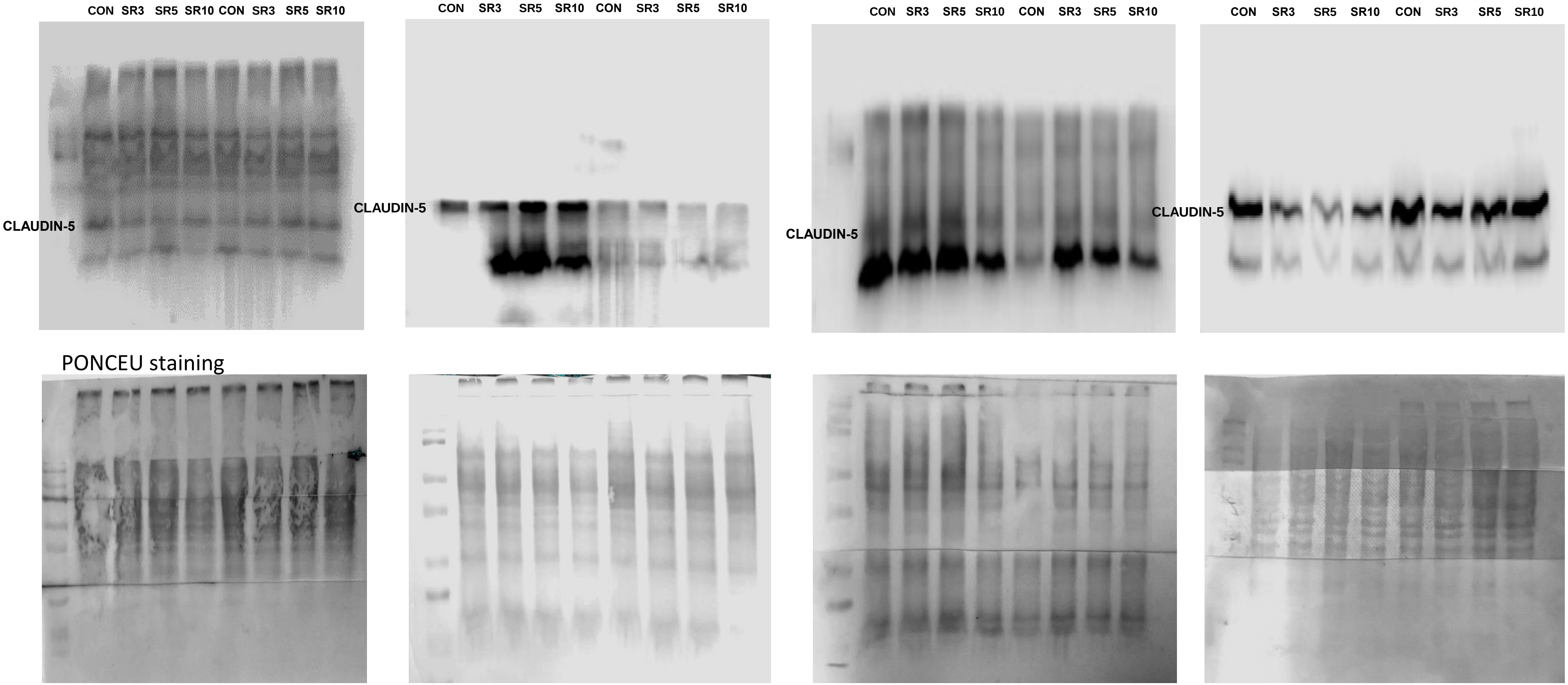

Cerebral cortex: Claudin-5

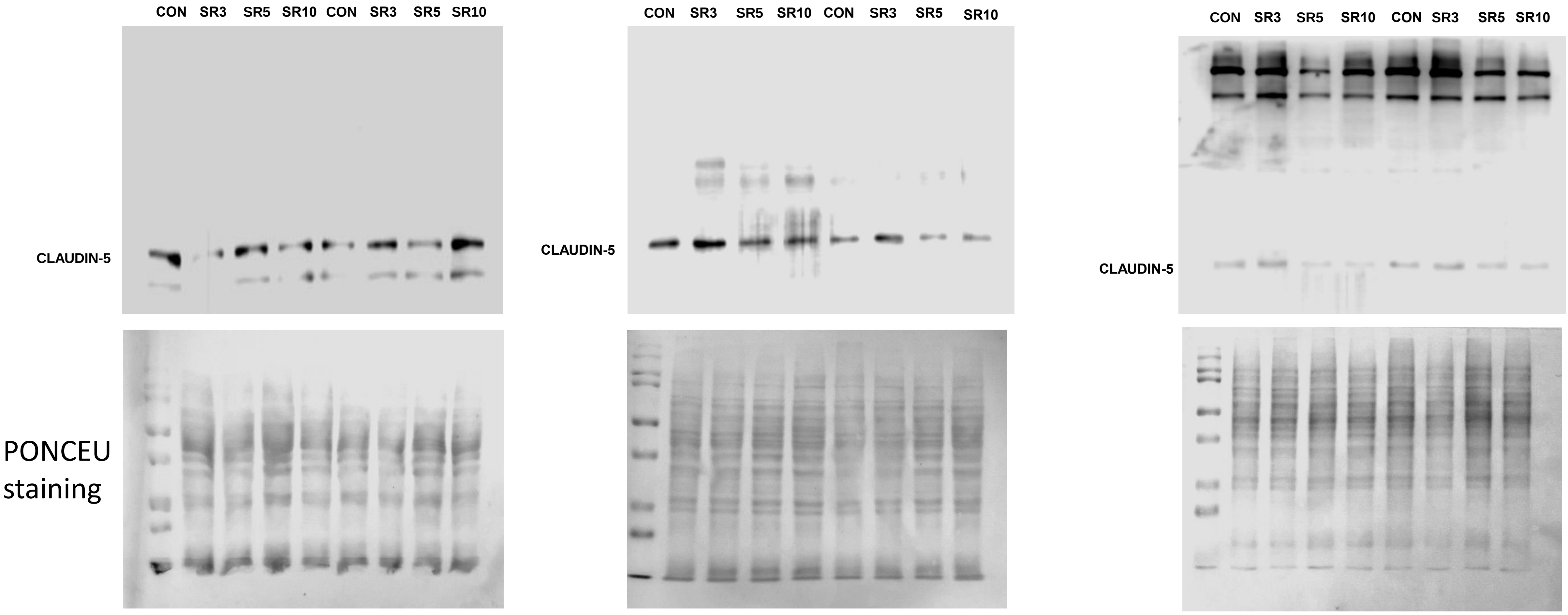

CEREBRAL CORTEX: ZO-1

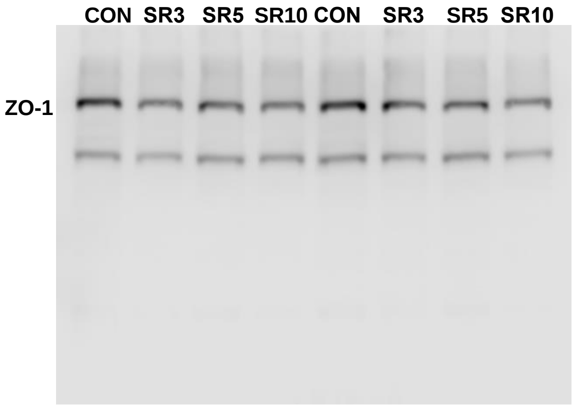

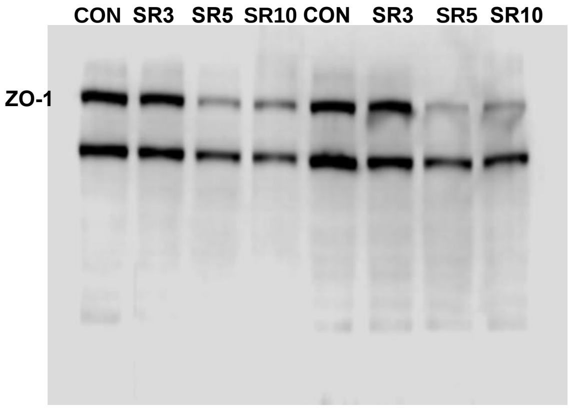

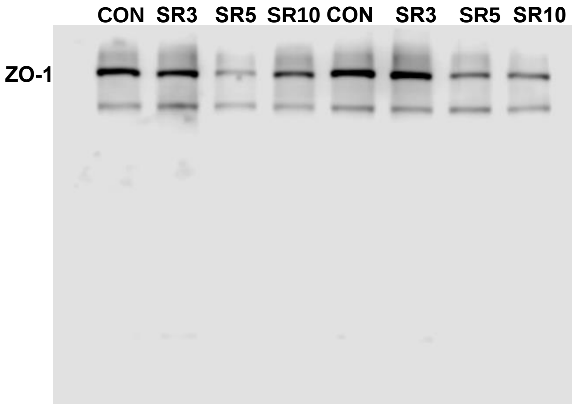

PONCEU staining

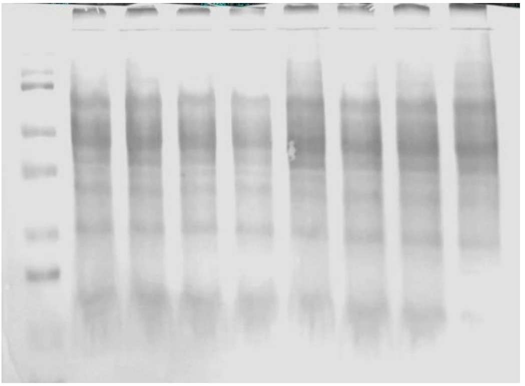

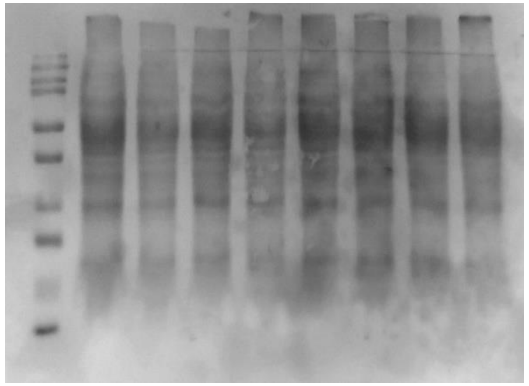

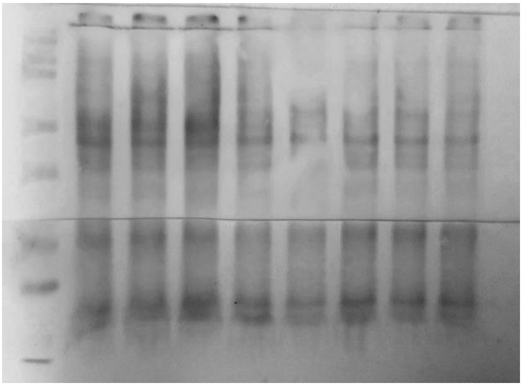

CEREBRAL CORTEX: ZO-1

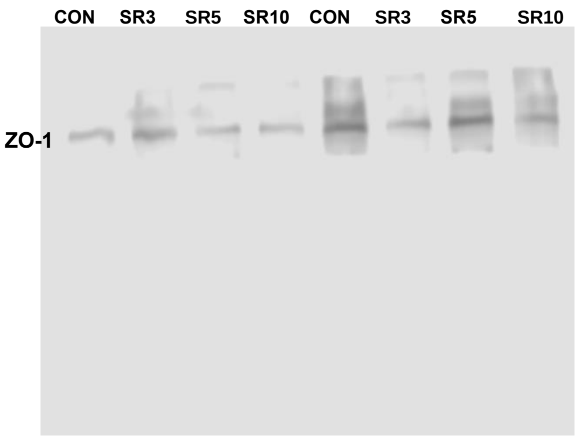

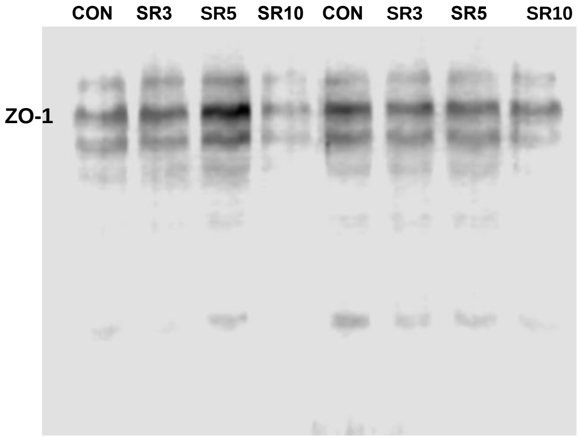

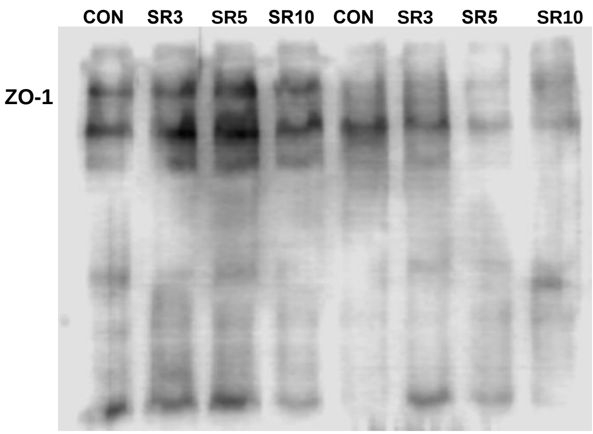

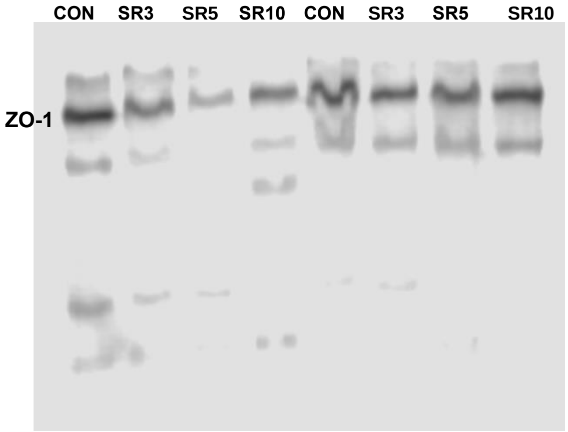

PONCEU staining

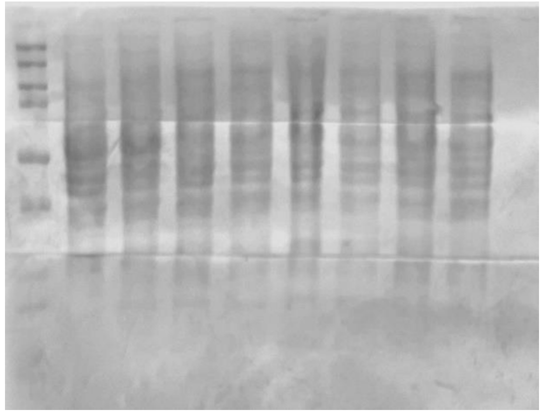

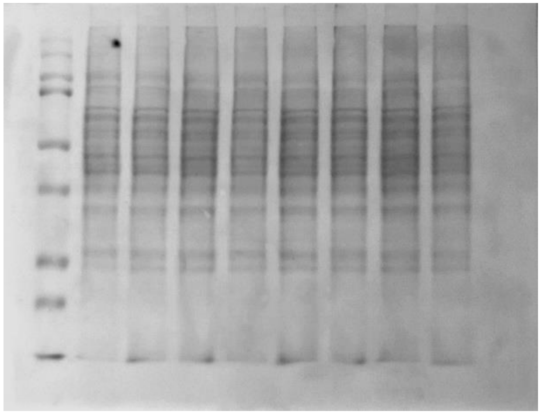

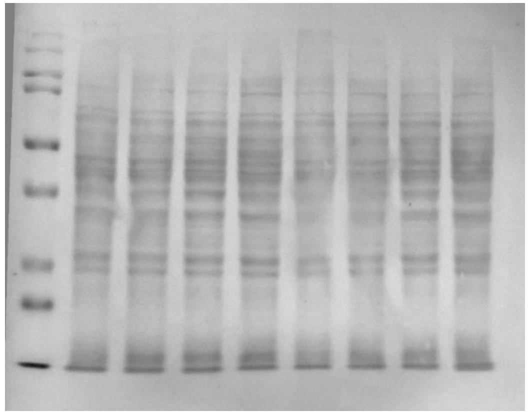

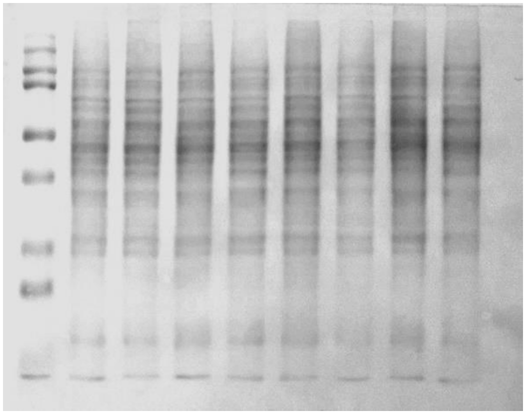

CEREBRAL CORTEX: GFAP

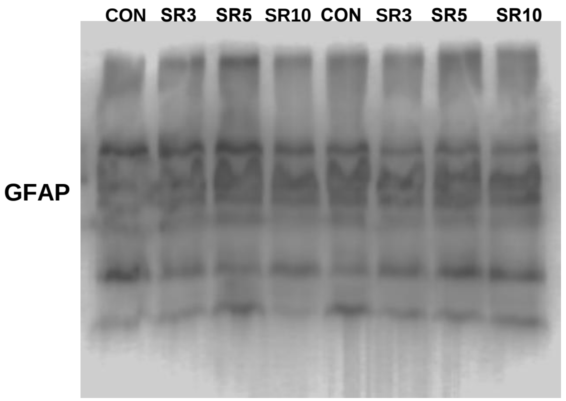

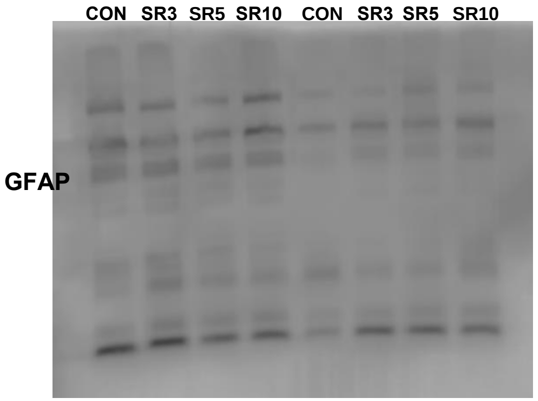

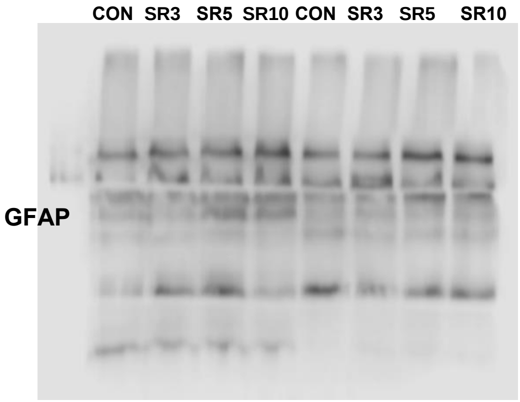

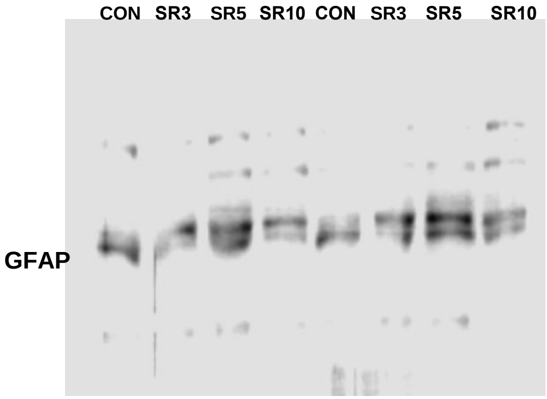

PONCEU staining

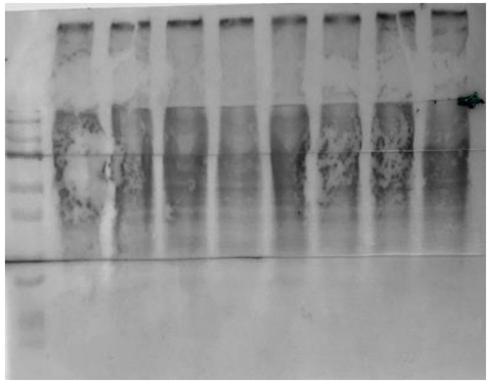

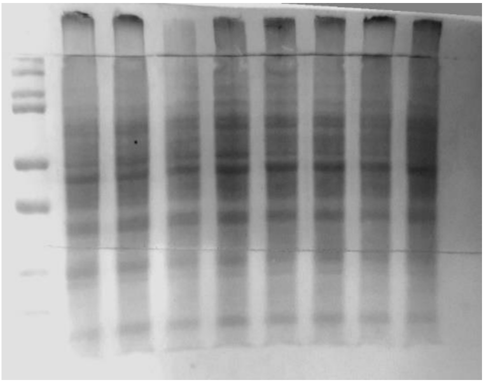

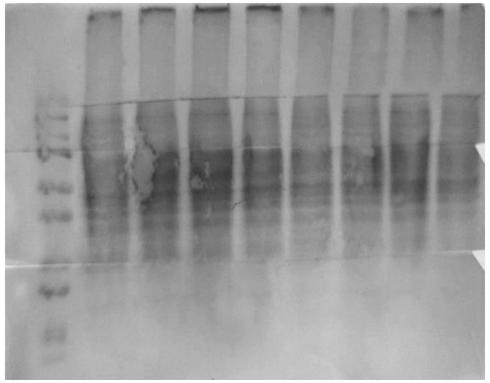

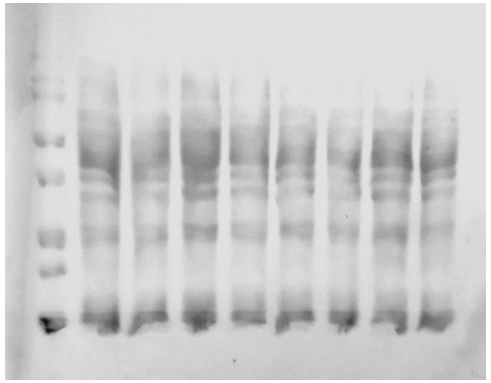

CEREBRAL CORTEX: GFAP

CON SR3 SR5 SR10 CON SR3 SR5 SR10

GFAP

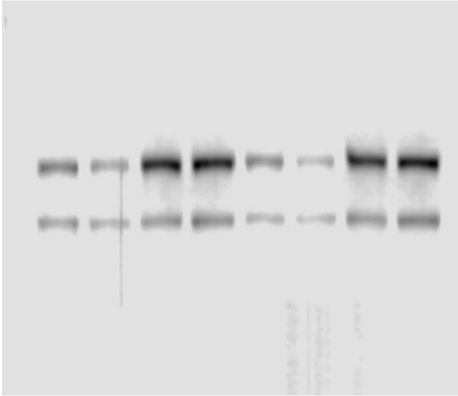

CON SR3 SR5 SR10 CON SR3 SR5 SR10

GFAP

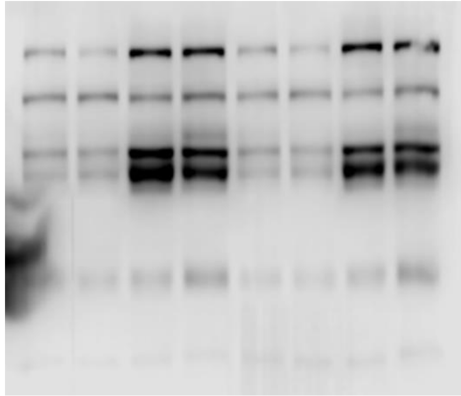

CON SR3 SR5 SR10 CON SR3 SR5 SR10

GFAP

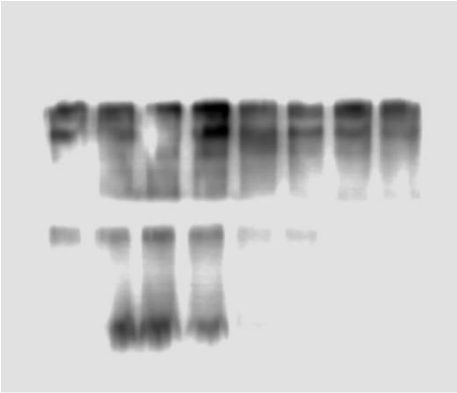

PONCEU staining

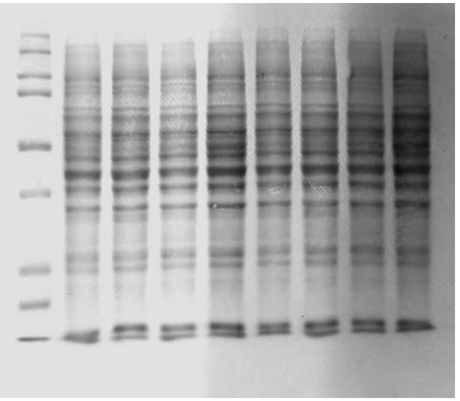

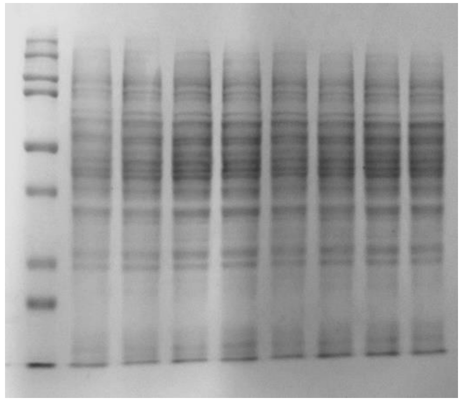

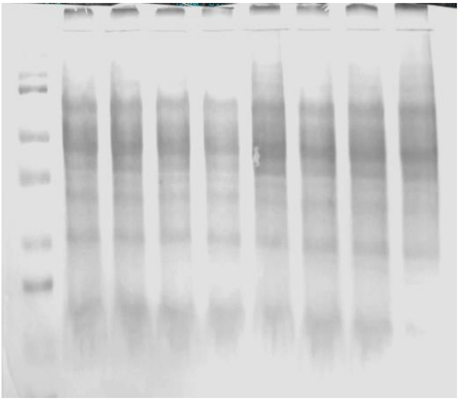

CEREBRAL CORTEX: C3

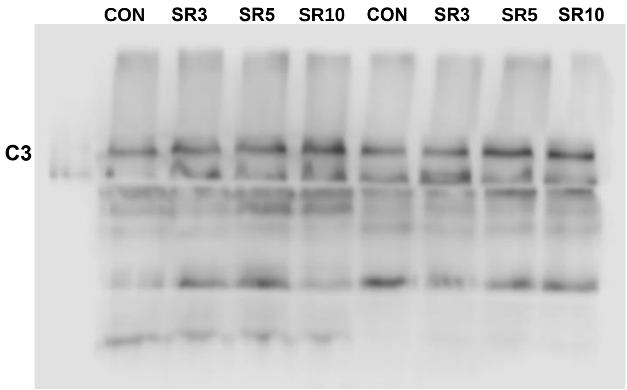

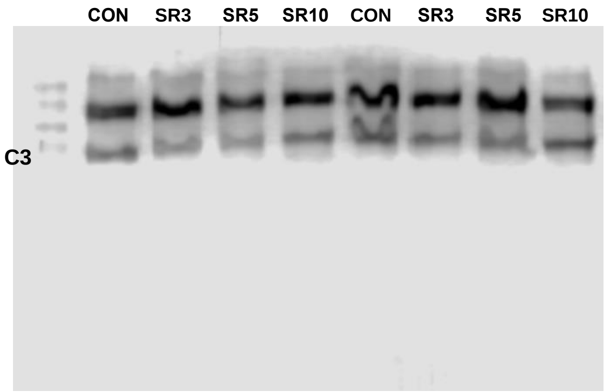

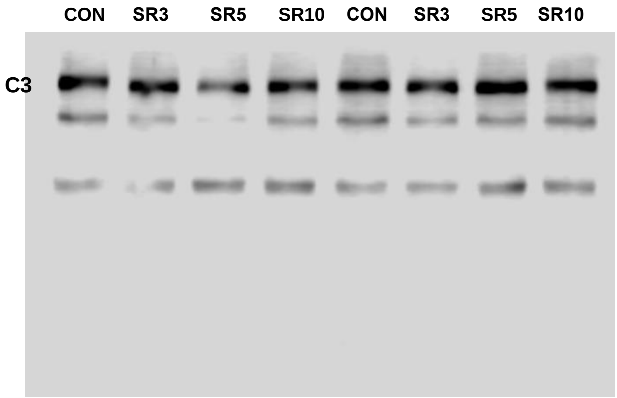

PONCEU staining

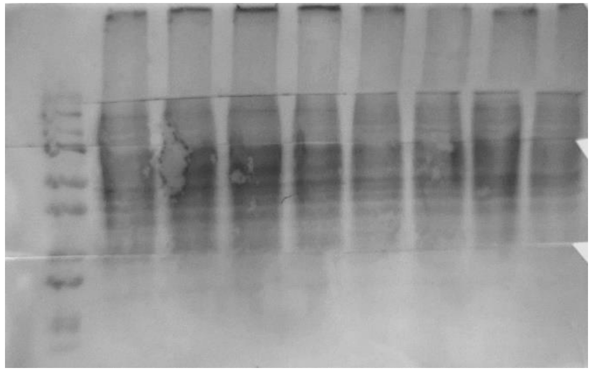

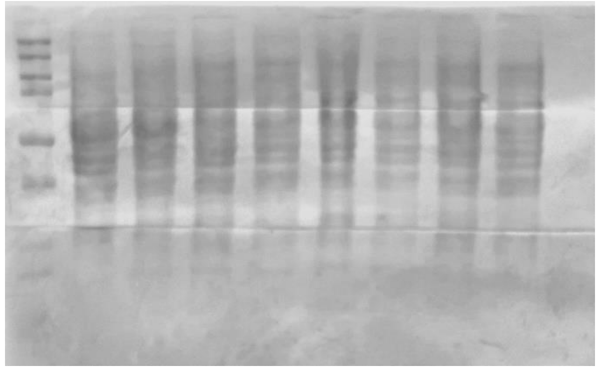

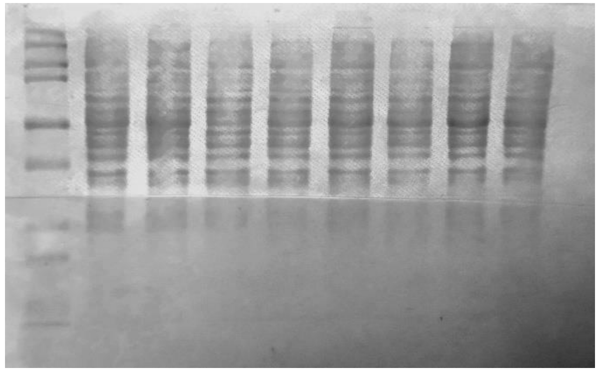

CEREBRAL CORTEX: C3

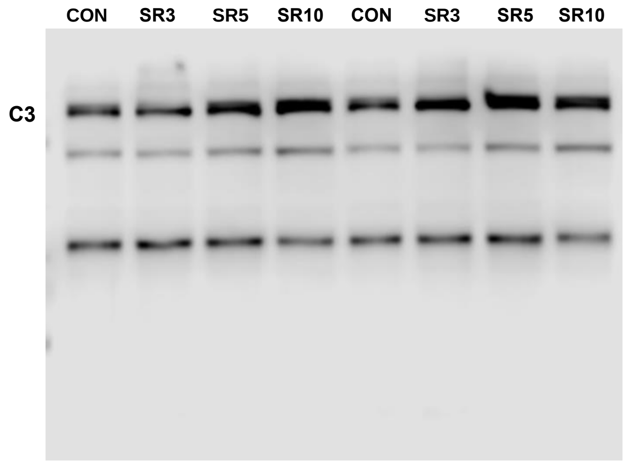

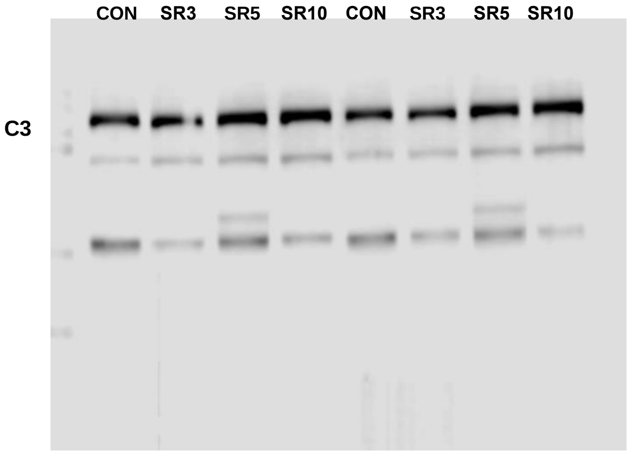

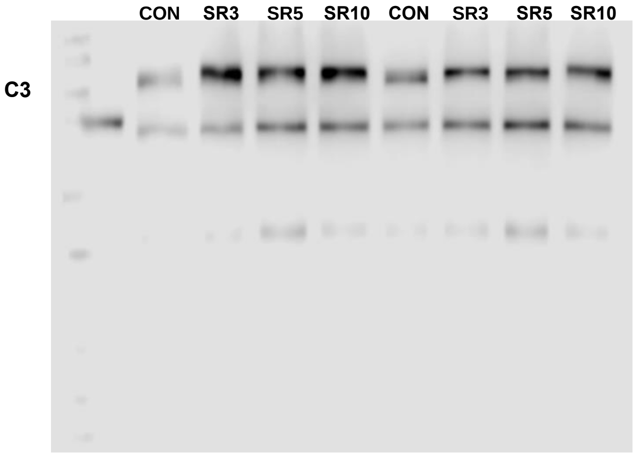

PONCEU staining

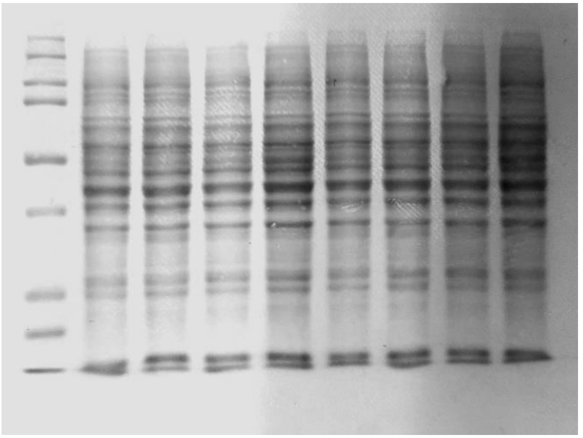

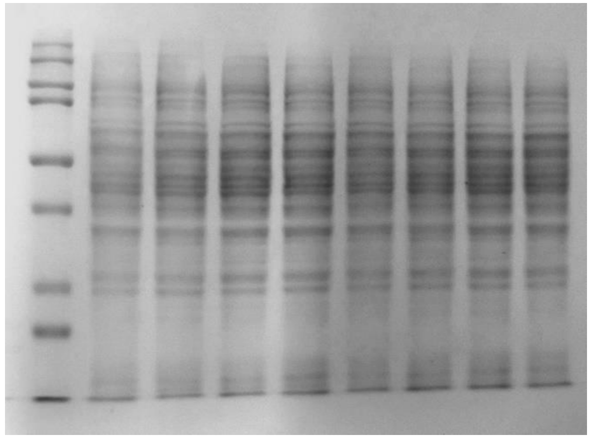

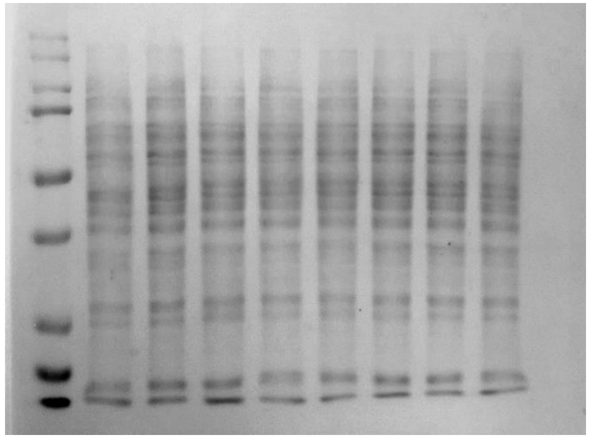

CEREBRAL CORTEX: S100a10

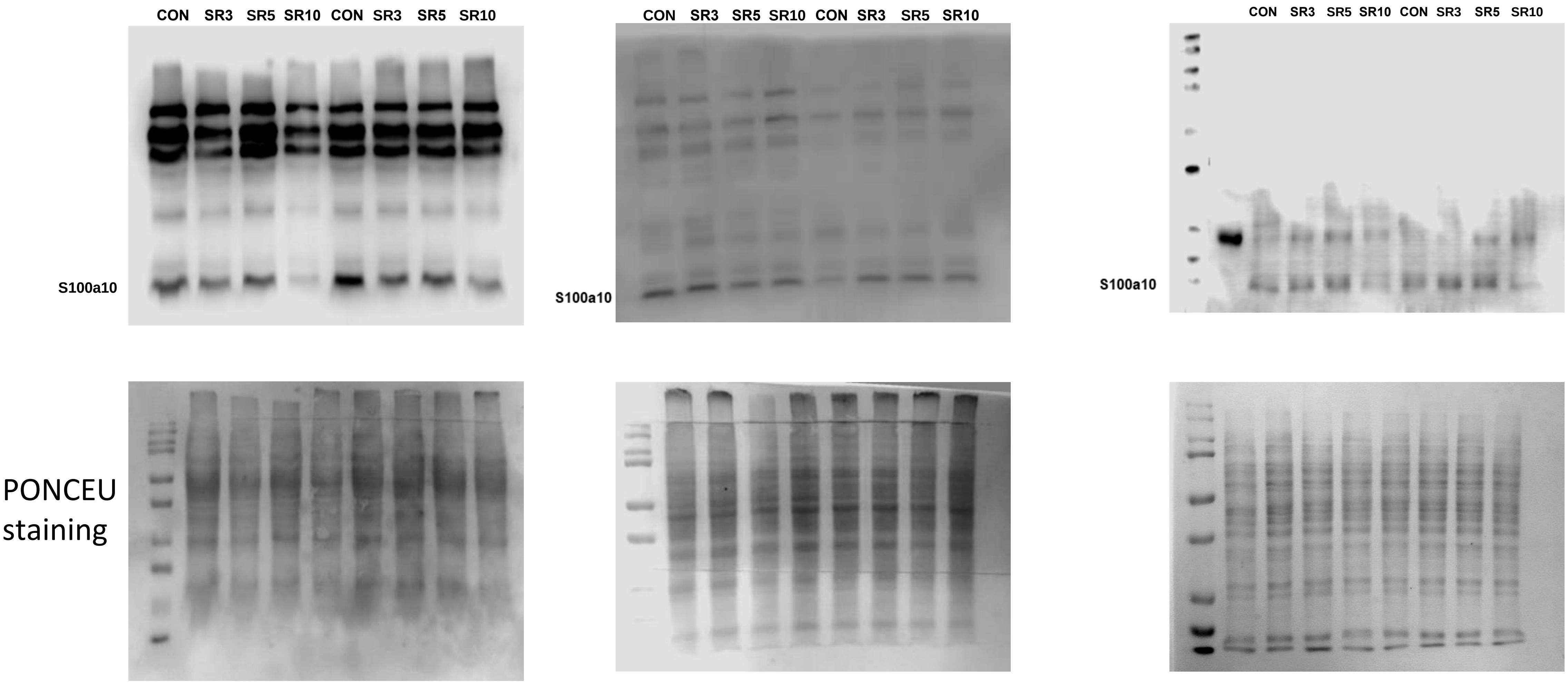

CEREBRAL CORTEX: S100a10

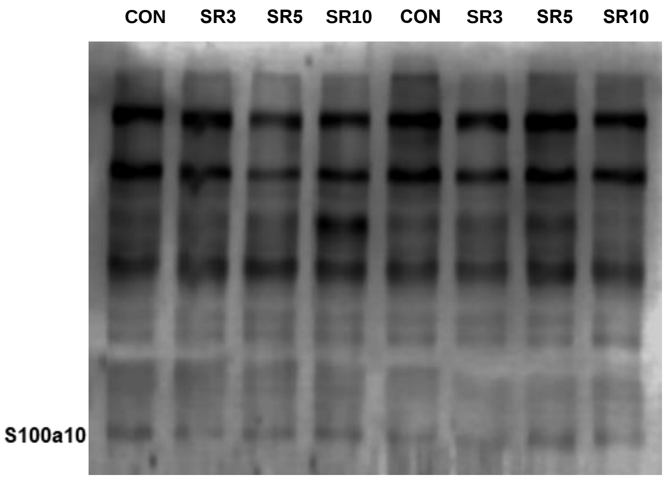

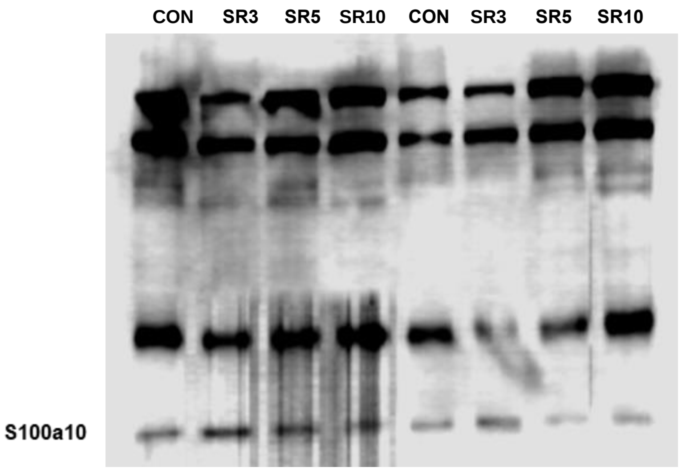

PONCEU staining

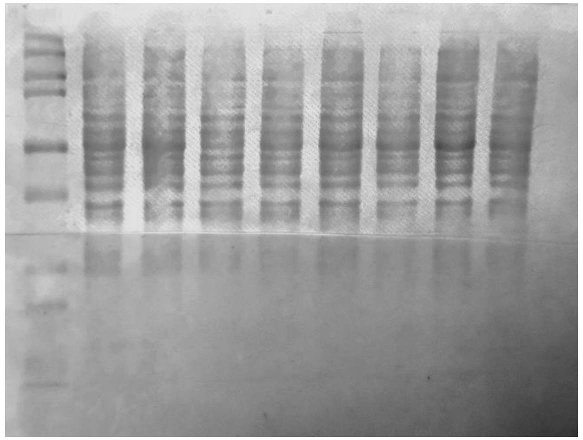

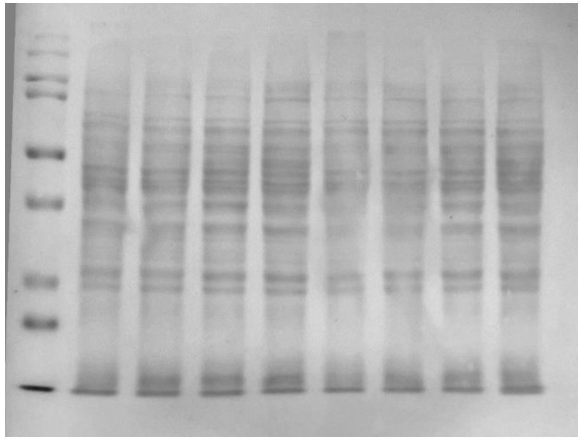

Cerebral cortex: β-galactosidase

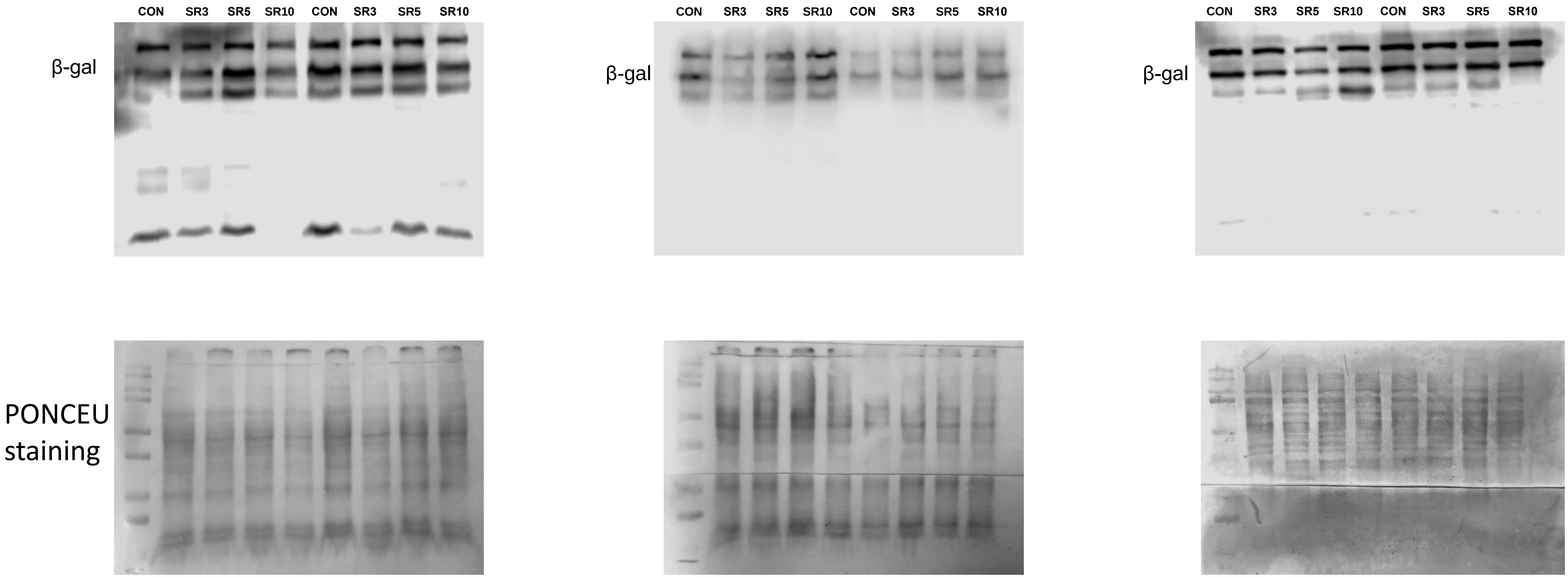

Cerebral cortex: β-galactosidase

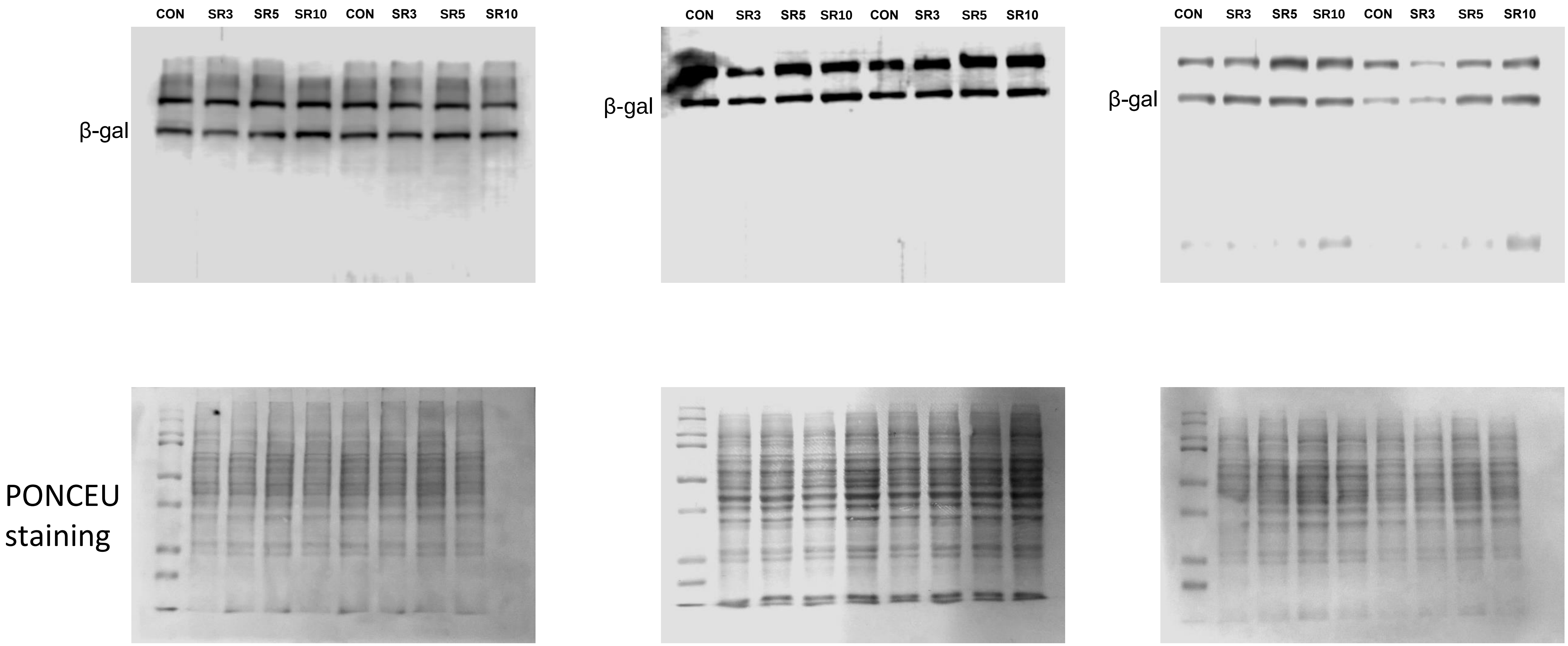

Cerebral cortex: p21

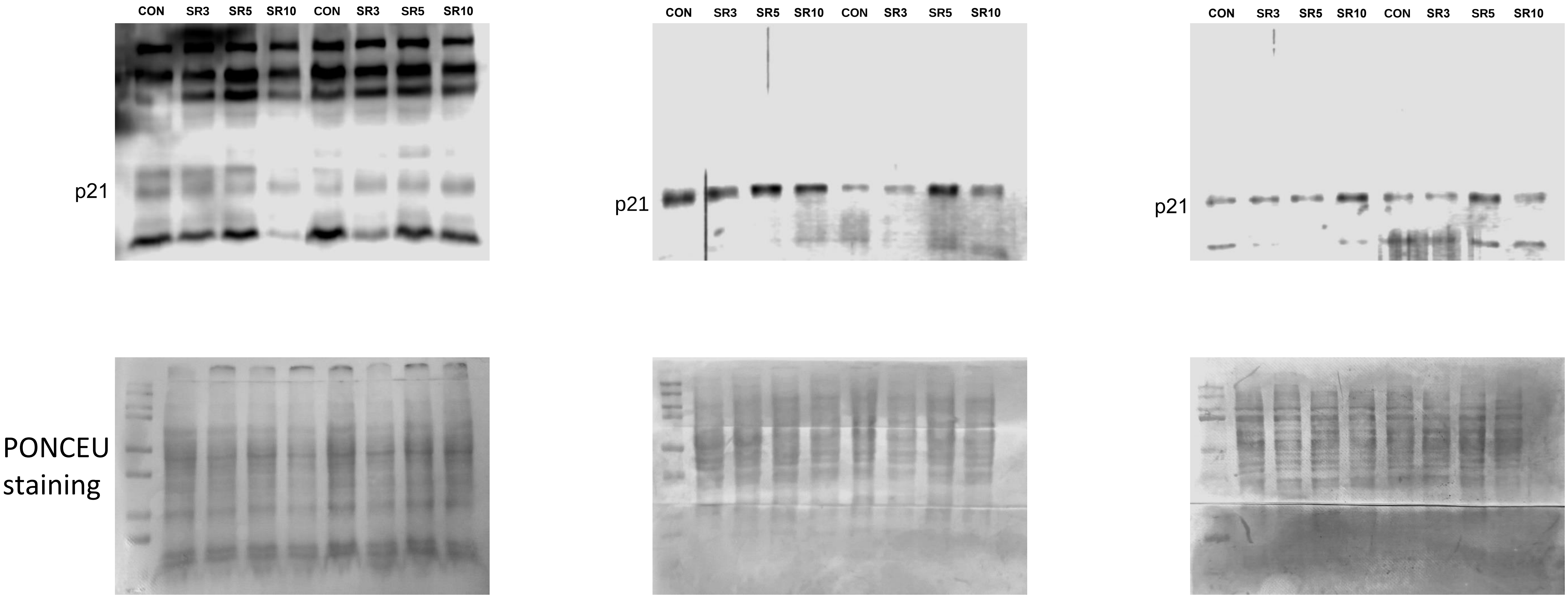

Cerebral cortex: p21

CON SR3 SR5 SR10 CON SR3 SR5 SR10

p21

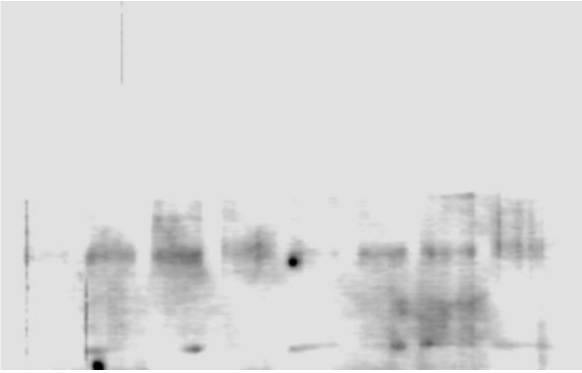

CON SR3 SR5 SR10 CON SR3 SR5 SR10

p21

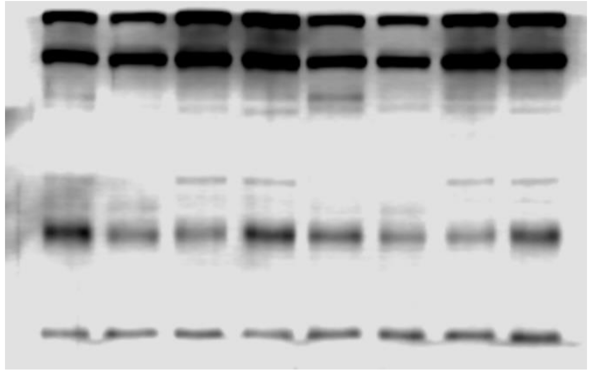

CON SR3 SR5 SR10 CON SR3 SR5 SR10

p21

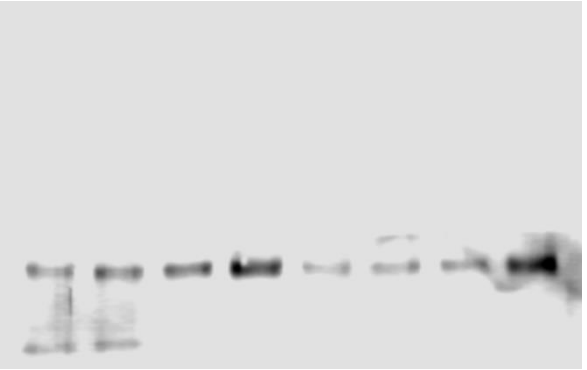

PONCEU staining

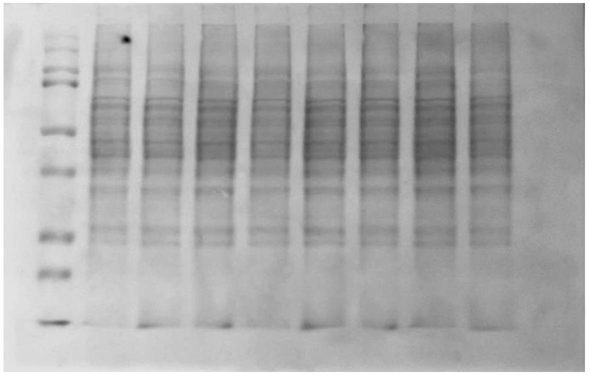

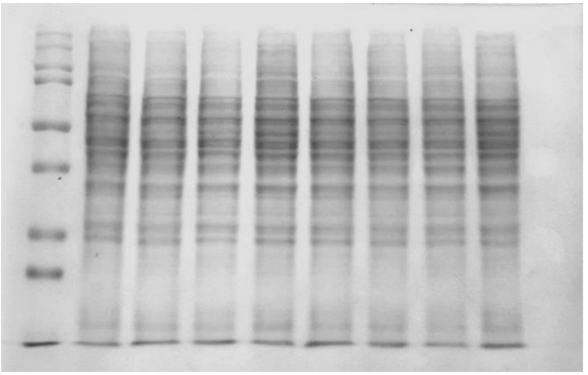

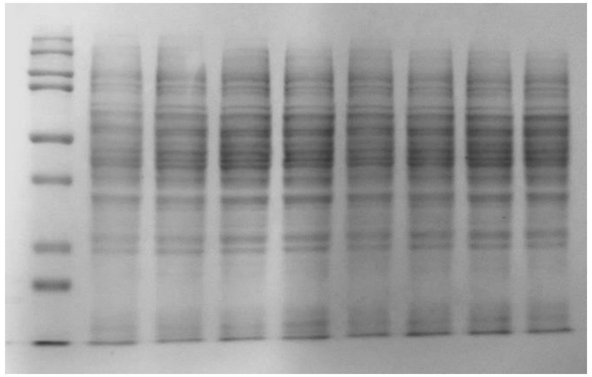

Hippocampus: Claudin-5

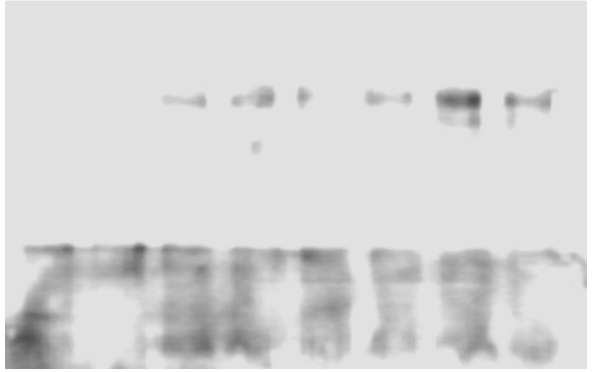

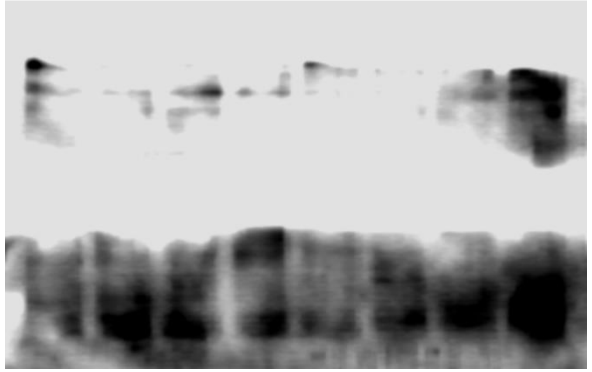

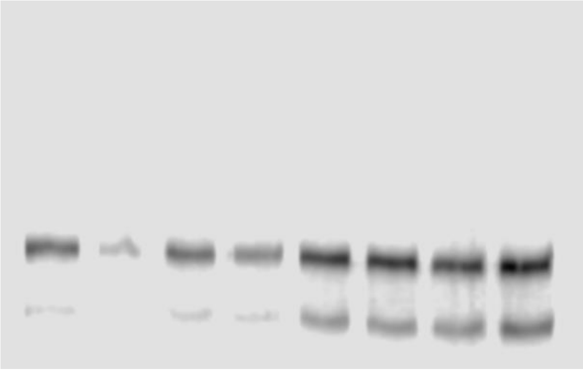

PONCEU staining

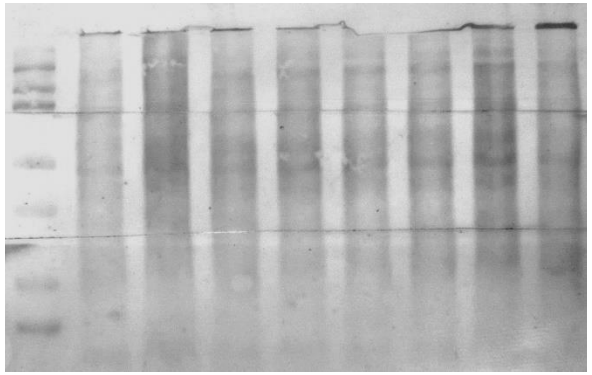

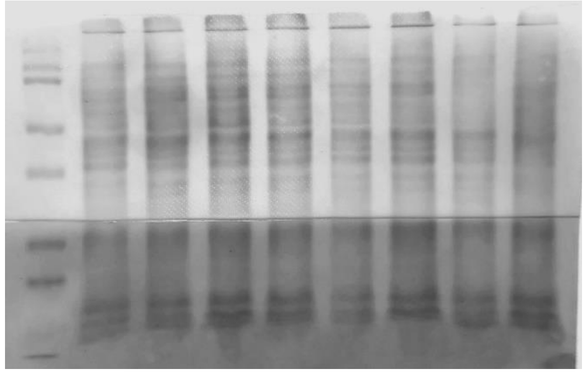

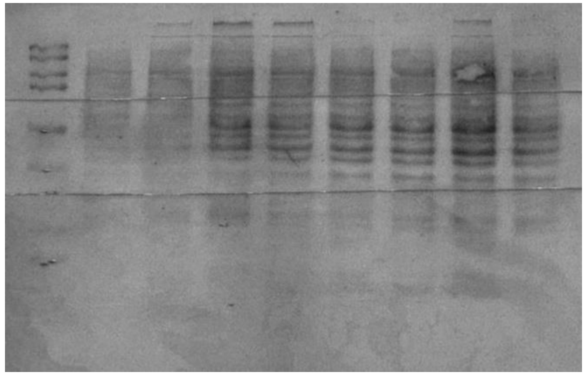

Hippocampus: Claudin-5

CON SR3 SR5 SR10 CON SR3 SR5 SR10

CLAUDIN-5

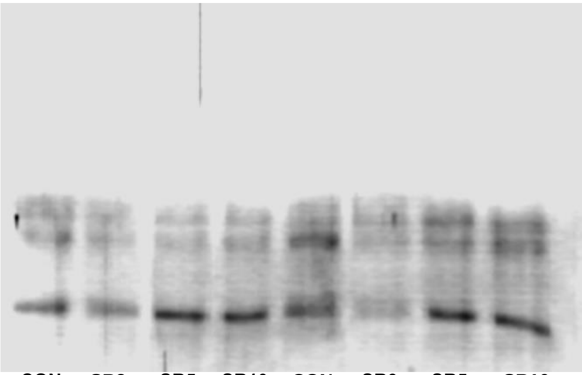

CON SR3 SR5 SR10 CON SR3 SR5 SR10

CON SR3 SR5 SR10 CON SR3 SR5 SR10

CLAUDIN-5

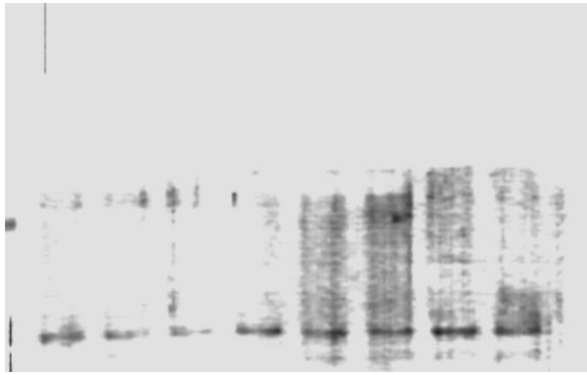

PONCEU
staining

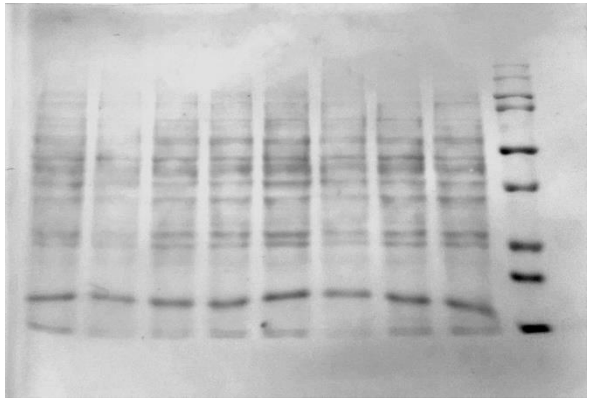

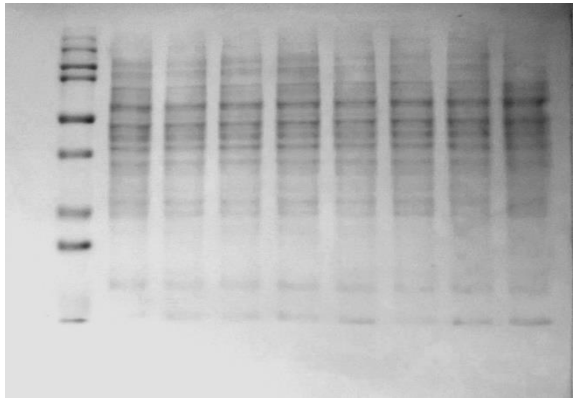

Hippocampus: ZO-1

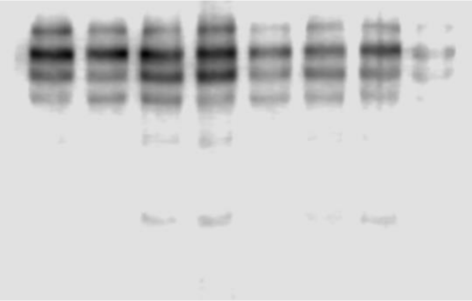

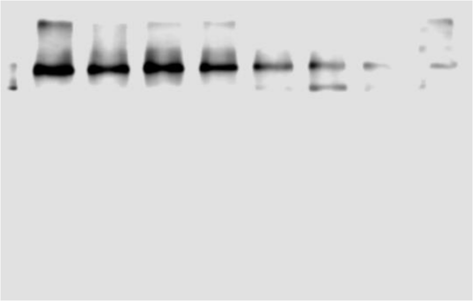

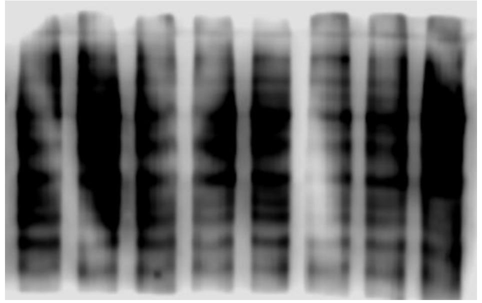

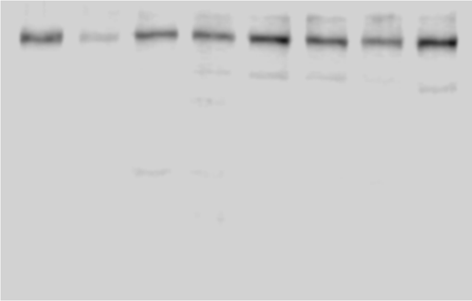

PONCEU staining

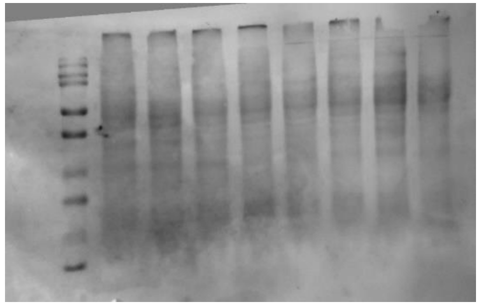

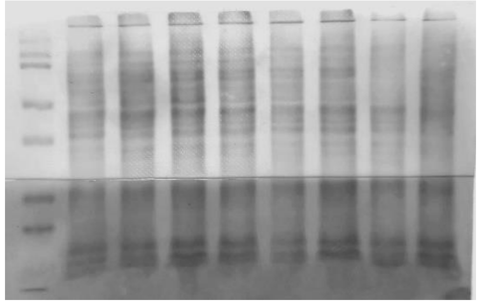

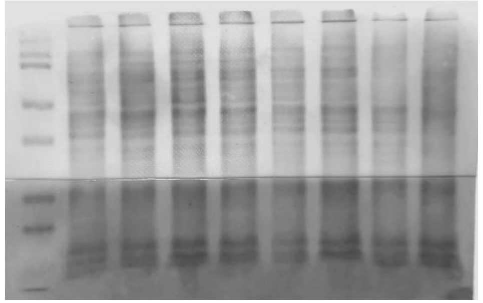

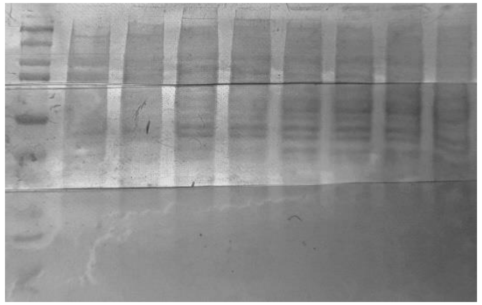

Hippocampus: ZO-1

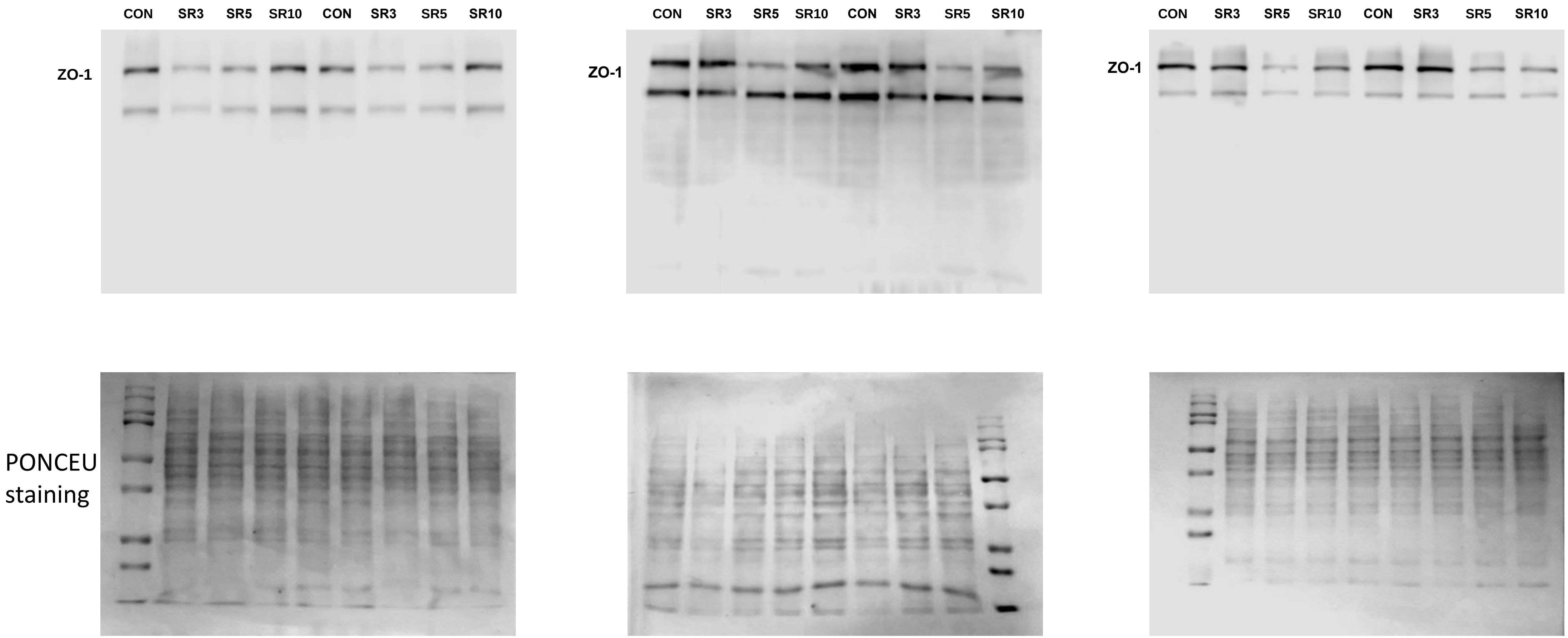

Hippocampus : GFAP

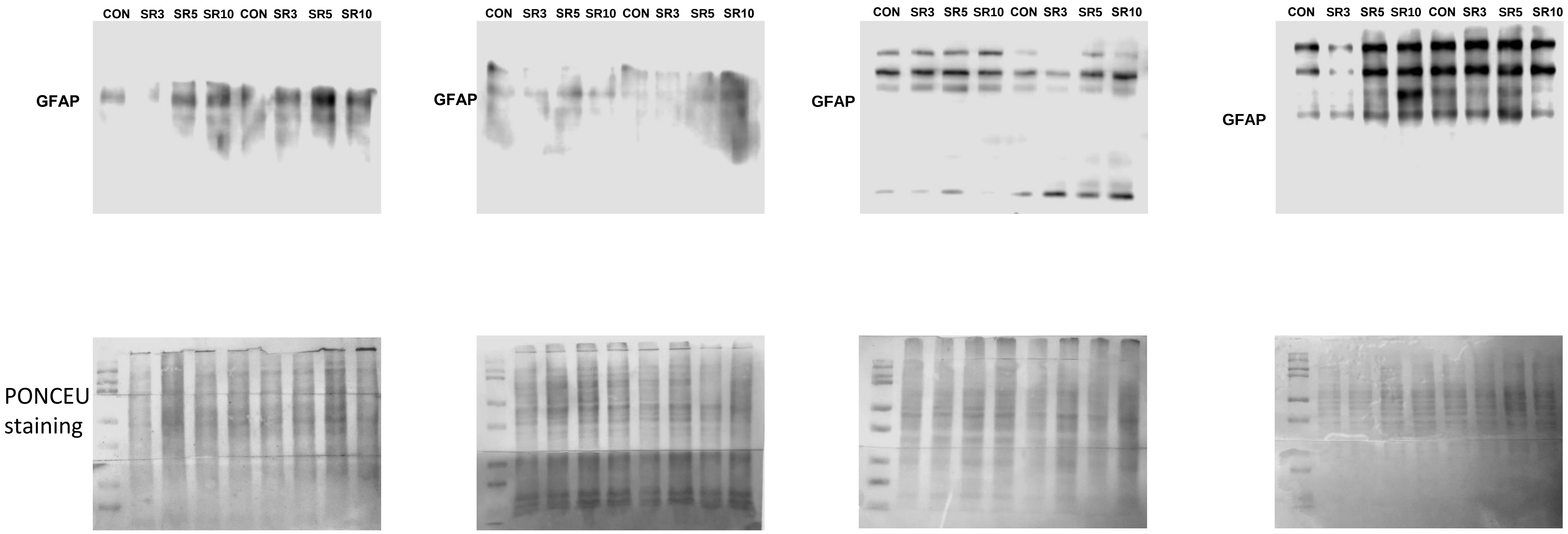

Hippocampus : GFAP

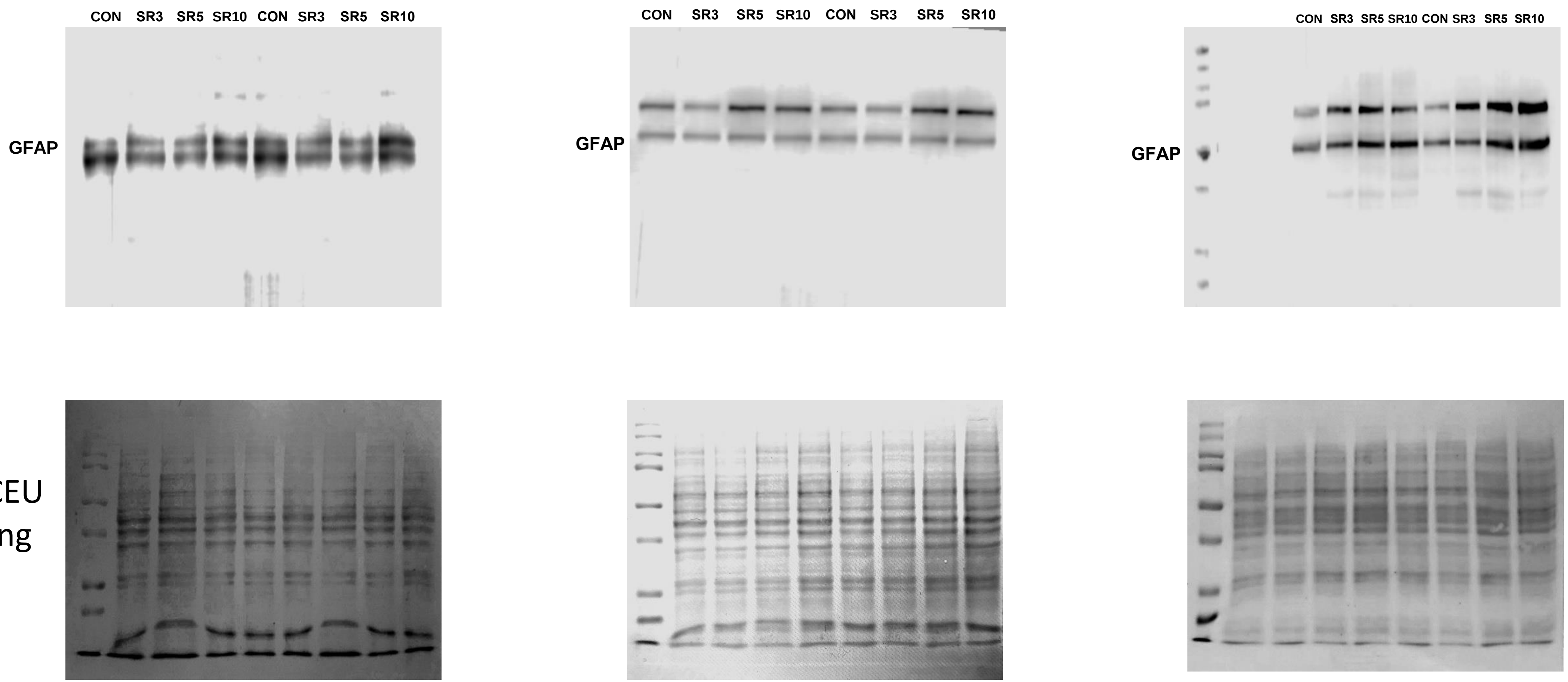

PONCEU staining

Hippocampus : C3

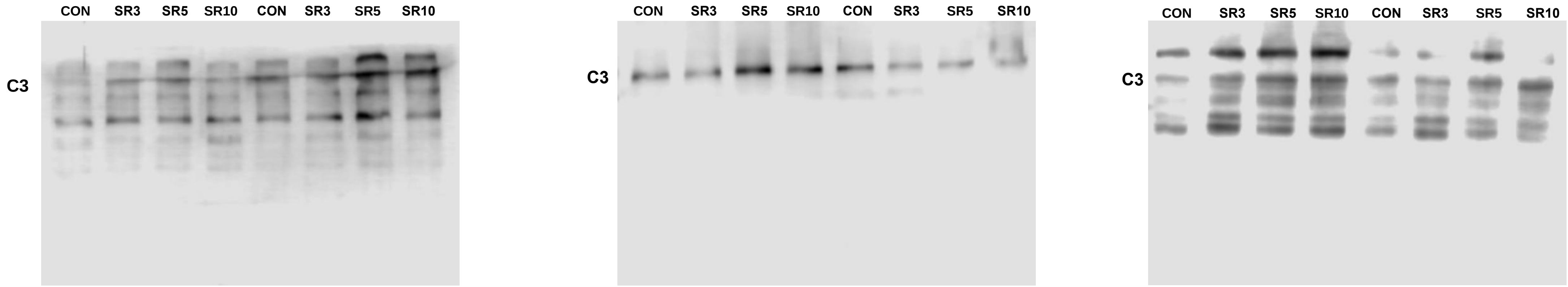

PONCEU staining

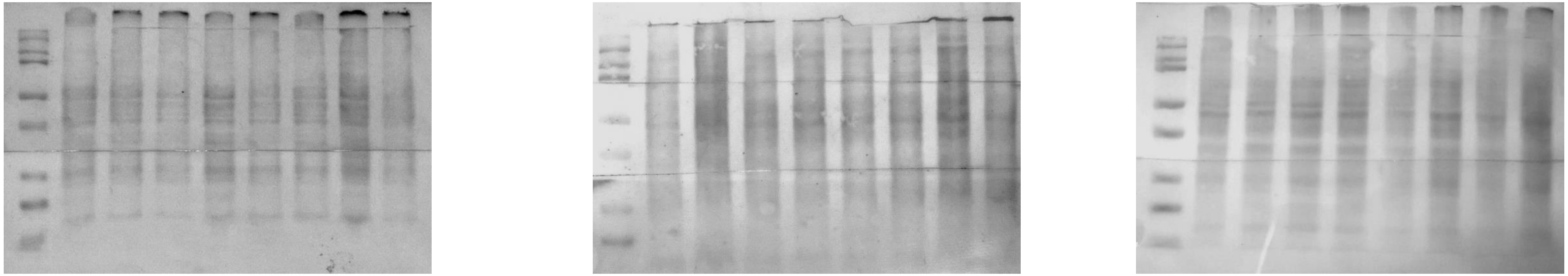

Hippocampus : C3

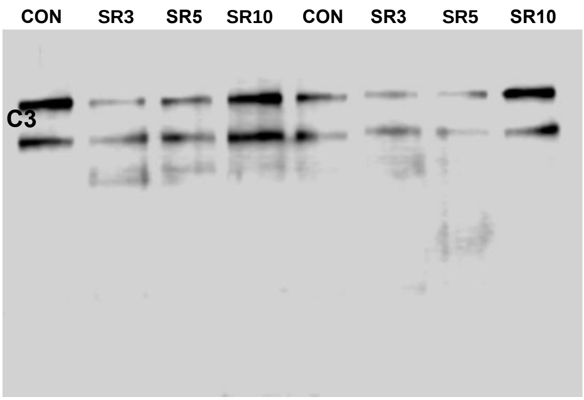

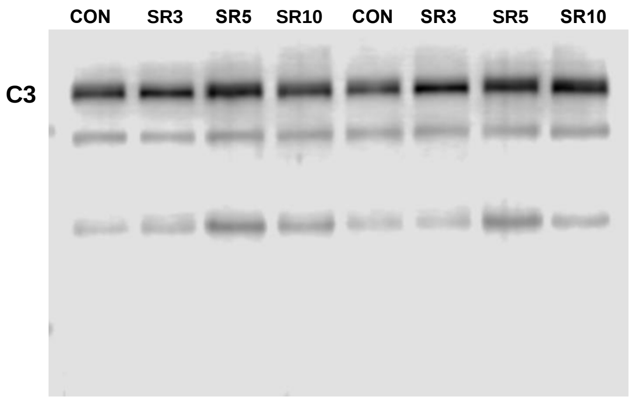

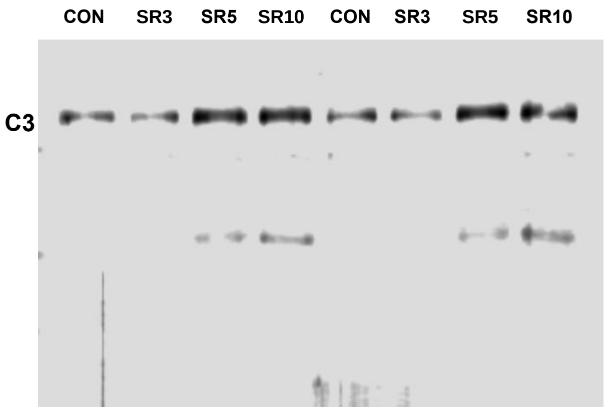

PONCEU staining

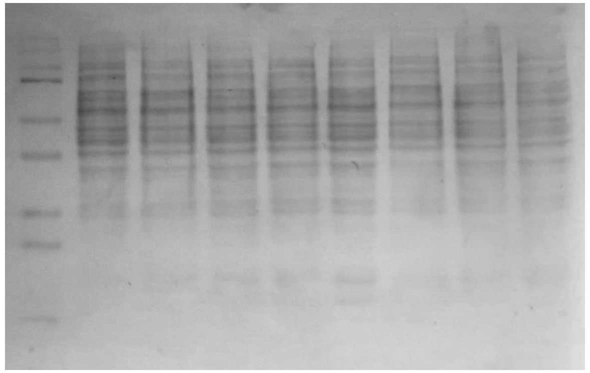

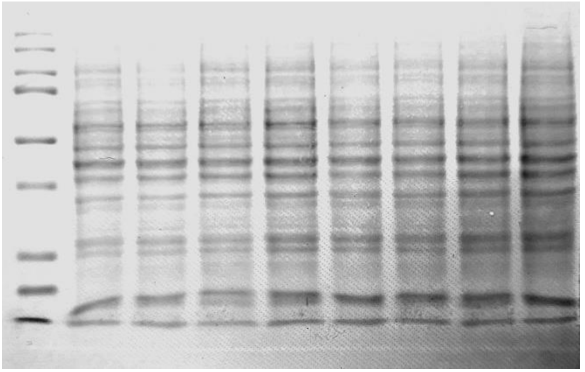

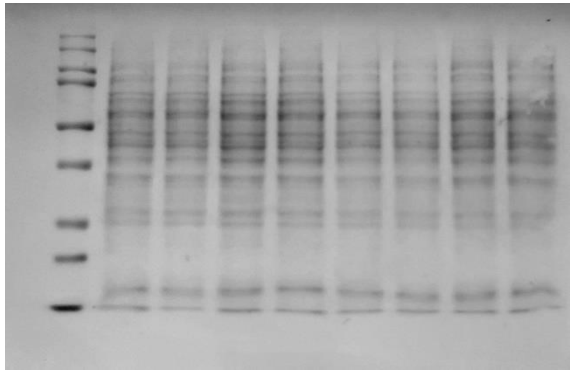

Hippocampus: S100a10

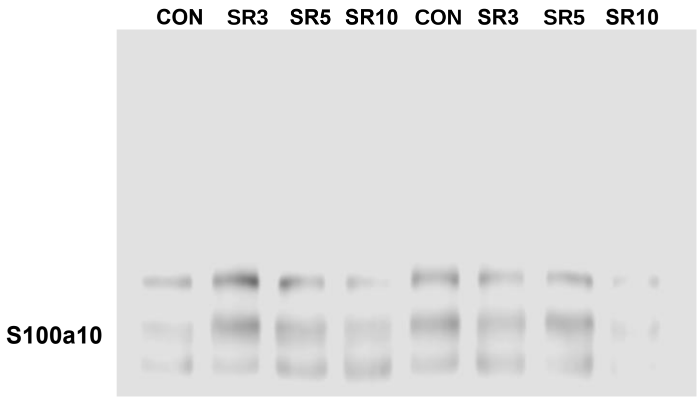

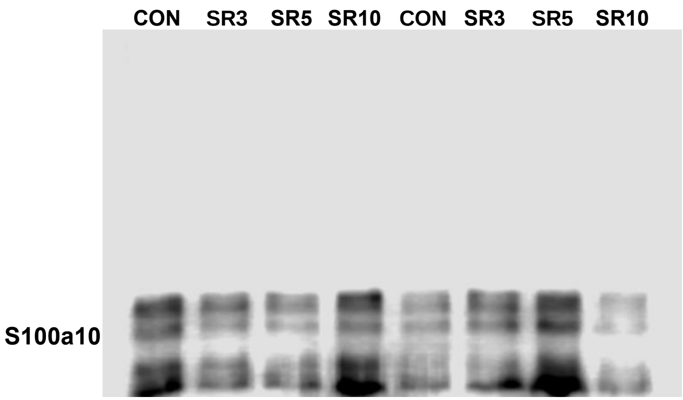

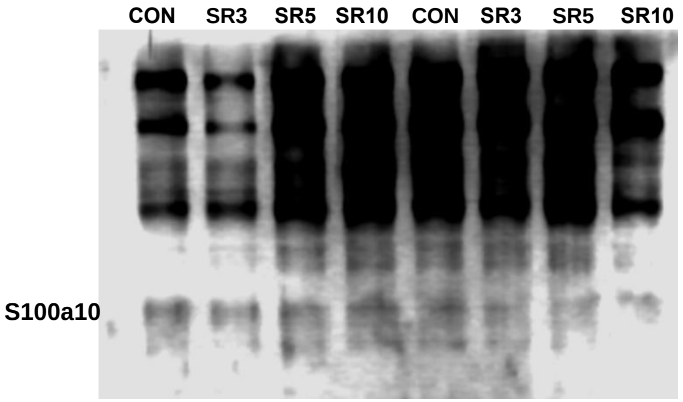

PONCEU staining

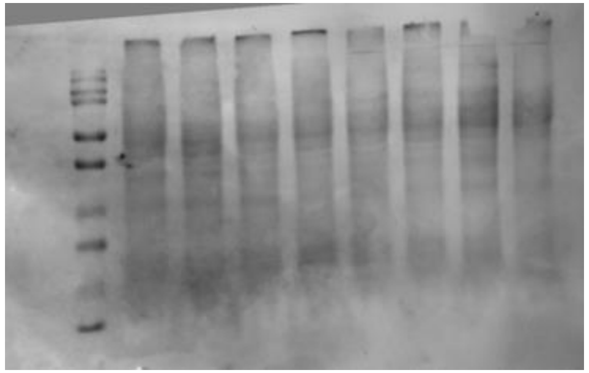

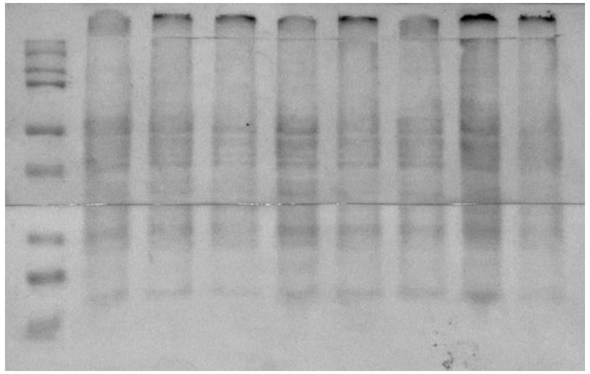

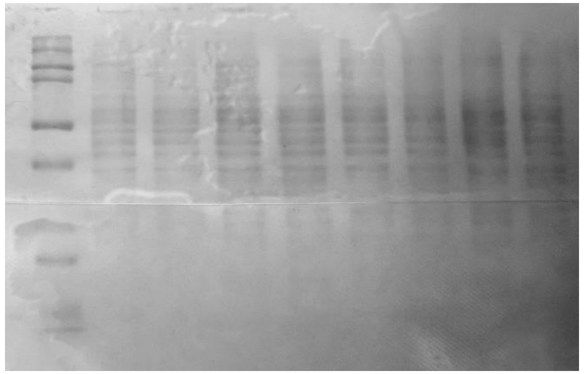

Hippocampus: S100a10

CON SR3 SR5 SR10 CON SR3 SR5 SR10

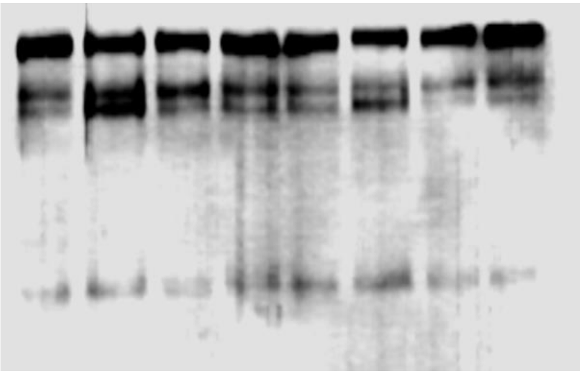

S100a10

CON SR3 SR5 SR10 CON SR3 SR5 SR10

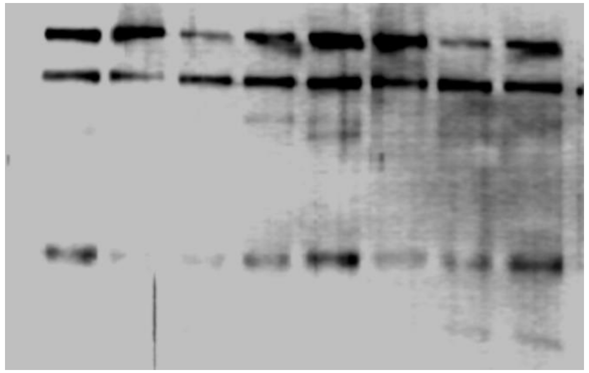

S100a10

CON SR3 SR5 SR10 CON SR3 SR5 SR10

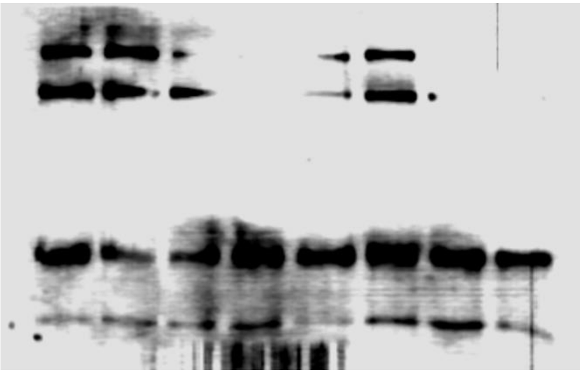

S100a10

PONCEU staining

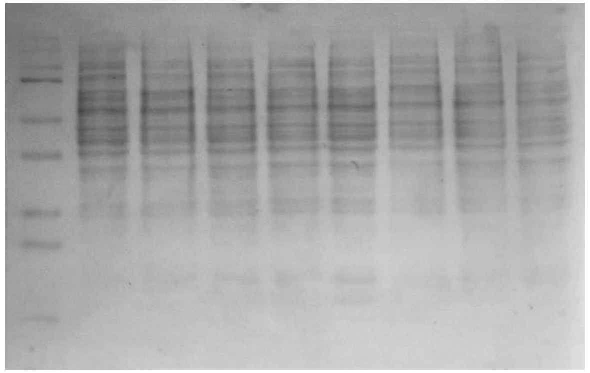

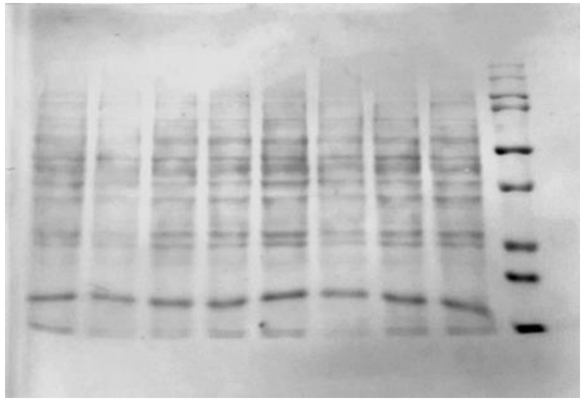

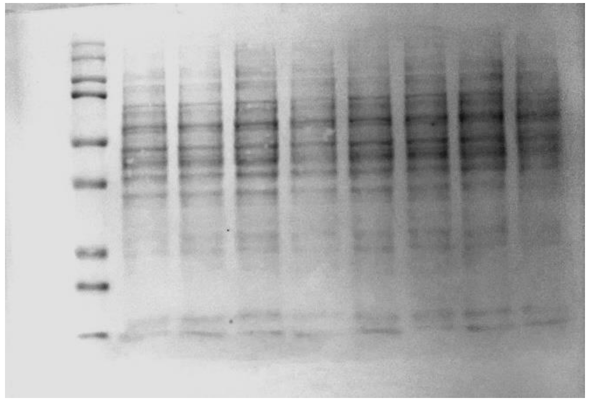

Hippocampus: β-galactosidase

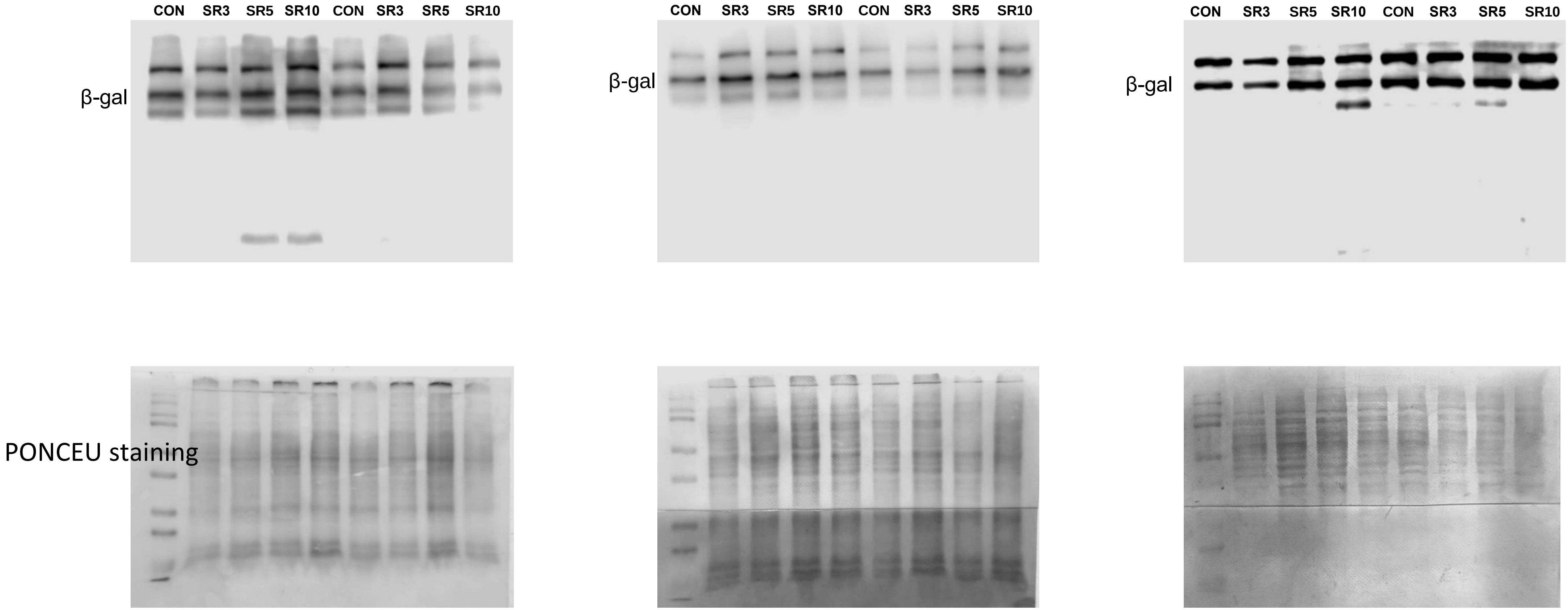

Hippocampus: β-galactosidase

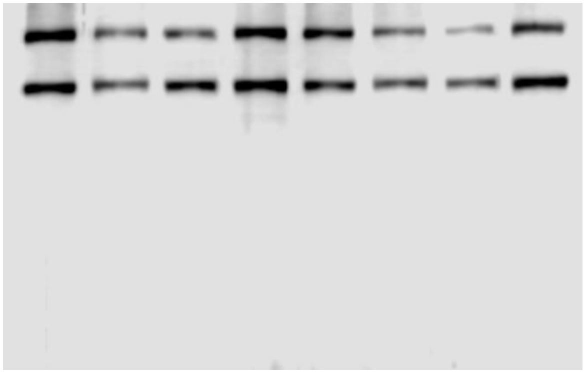

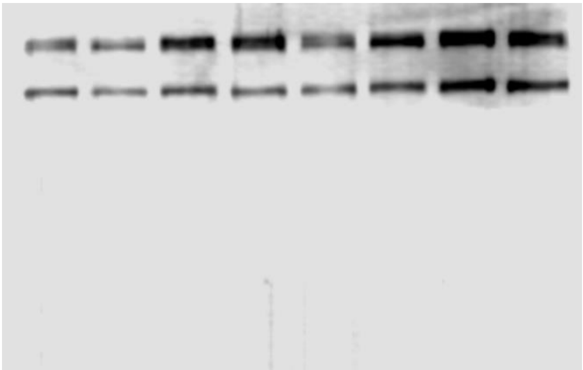

PONCEU staining

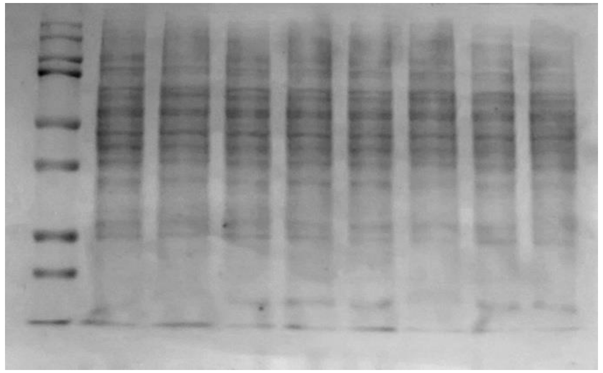

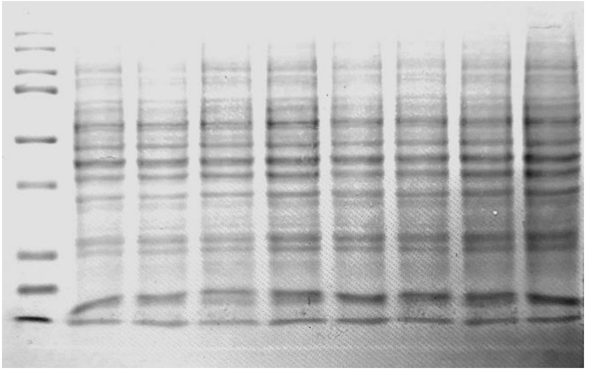

Hippocampus: p21

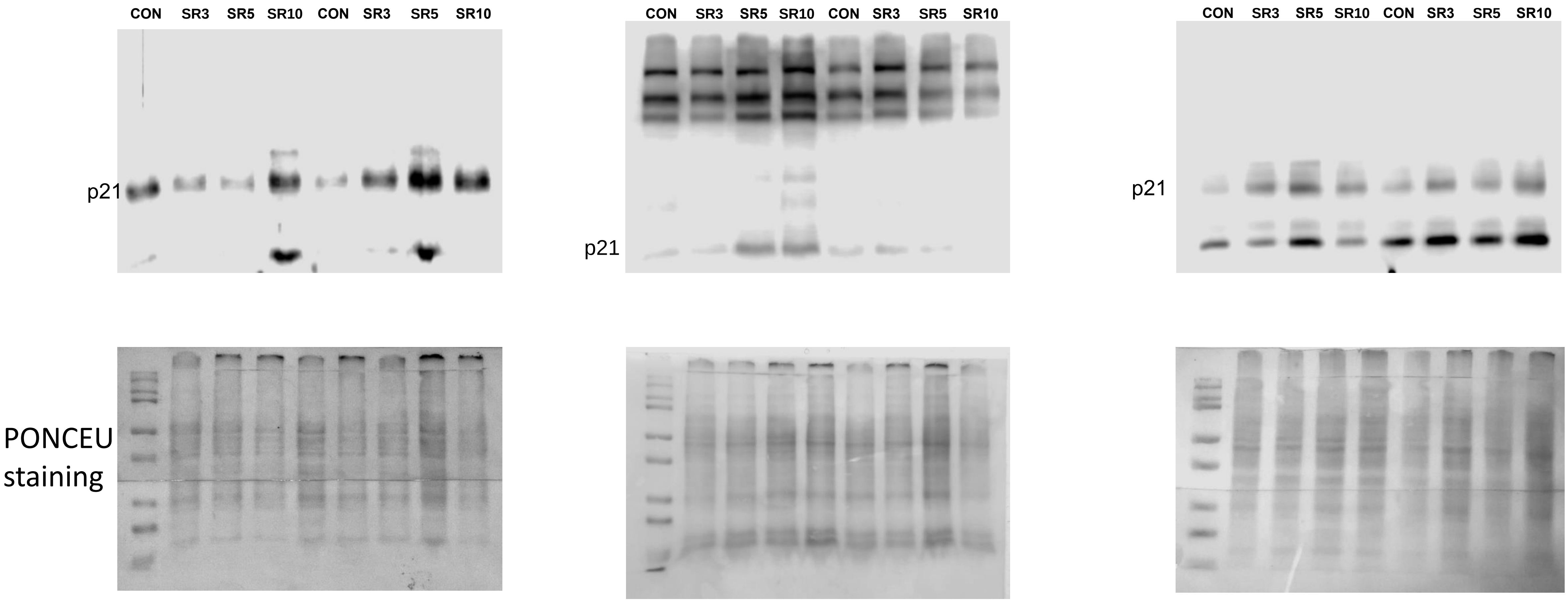

Hippocampus: p21

CON SR3 SR5 SR10 CON SR3 SR5 SR10

p21

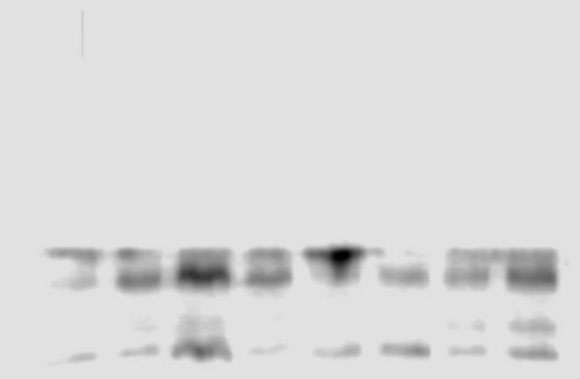

CON SR3 SR5 SR10 CON SR3 SR5 SR10

p21

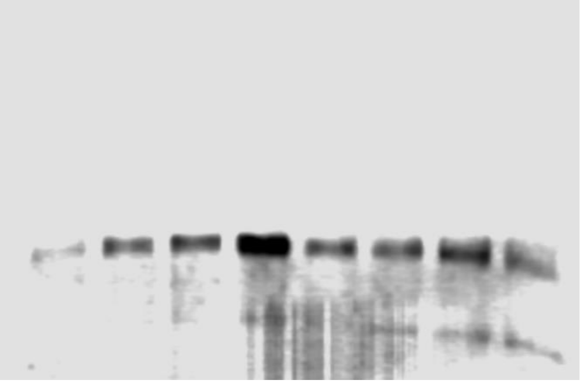

CON SR3 SR5 SR10 CON SR3 SR5 SR10

p21

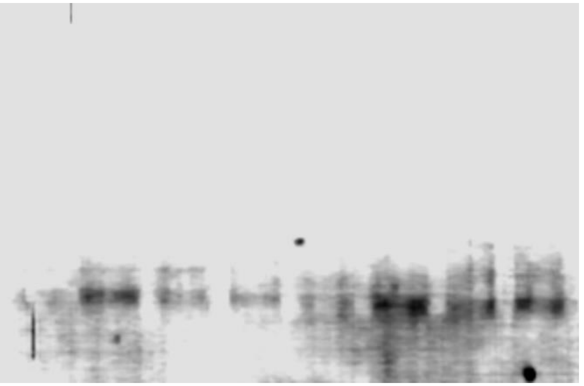

PONCEU staining

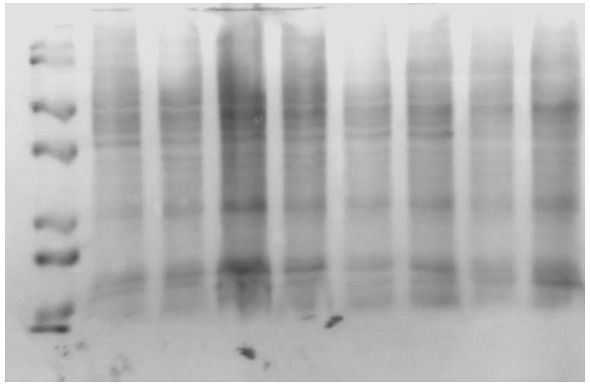

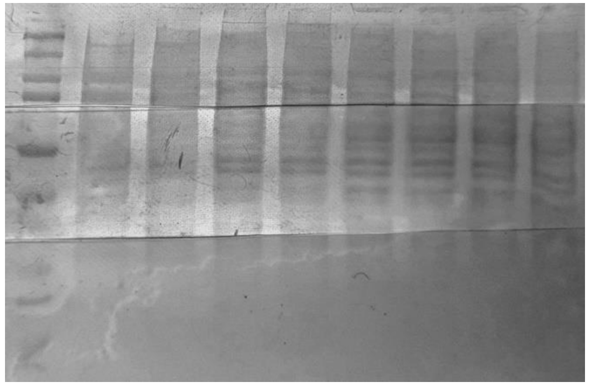

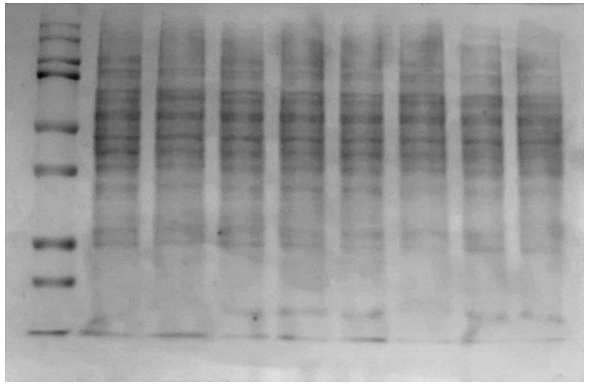

Supplement: Supplementary file 2 — Supplementary file2 (PDF 3038 KB) [file 11064_2025_4510_MOESM2_ESM.pdf]
