# Supplementary material for: Progressive Blood–Brain Barrier Disruption in Sleep-Restricted Young Mice: Cellular Senescence and Neuroinflammation Crosstalk
Source: Neurochem Res. 2025 Aug 18;50(5):269. doi: 10.1007/s11064-025-04510-y (PMC12361337; doi:10.1007/s11064-025-04510-y)
Supplement: Supplementary file 3 — Supplementary file3 (PDF 28421 KB) [file 11064_2025_4510_MOESM3_ESM.pdf]

BLOOD-BRAIN BARRIER DYSFUNCTION DURING SLEEP LOSS IS ASSOCIATED WITH CELLULAR SENESCENCE AND NEUROINFLAMMATION

Authors: Jessica J. Avilez-Avilez, J. Enrique García-Aviles, Ricardo Jair Ramírez-Carreto, Verónica Salas-Venegas, Mara A. Guzmán-Ruiz, Fernanda Medina-Flores, Mina Königsberg, Anahí Chavarria, Beatriz Gómez-González.

Fig. S1

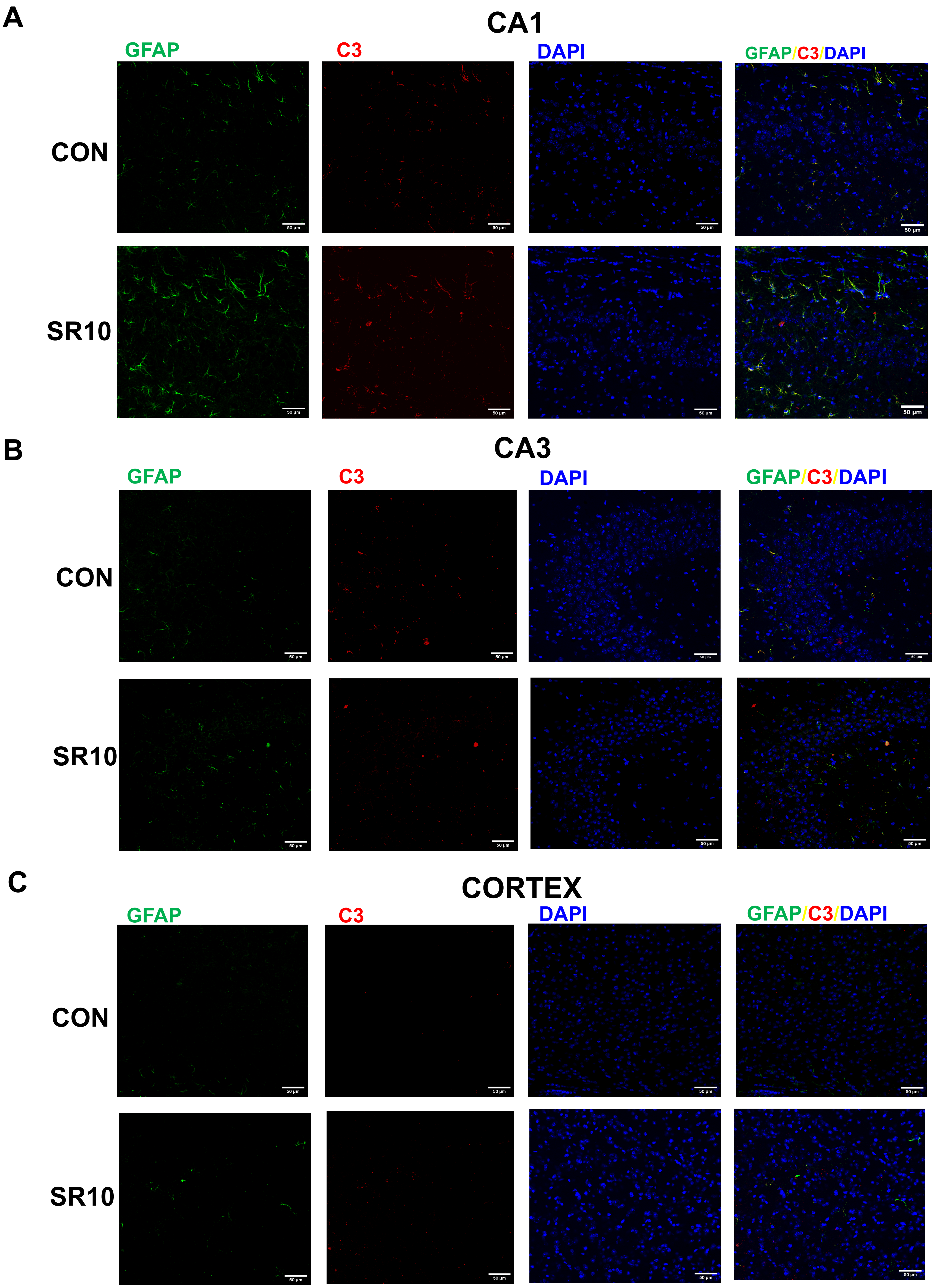
A
CA1
GFAP
C3
DAPI
GFAP/C3/DAPI
CON
SR10
50 μm
B
CA3
GFAP
C3
DAPI
GFAP/C3/DAPI
CON
SR10
50 μm
C
CORTEX
GFAP
C3
DAPI
GFAP/C3/DAPI
CON
SR10
50 μm

Fig. S1| C3 complement protein colocalized with the astrocytes. Representative images of Iba-1+C3 expression in the hippocampal regions (A) CA1, (B) CA3, and the (C) cerebral cortex from control (CON) or 10-day sleep-restricted (SR10) mice are shown. Merge and each label of Iba-1 (green), C3 (red), and DAPI (blue) are shown separately.

Fig. S2

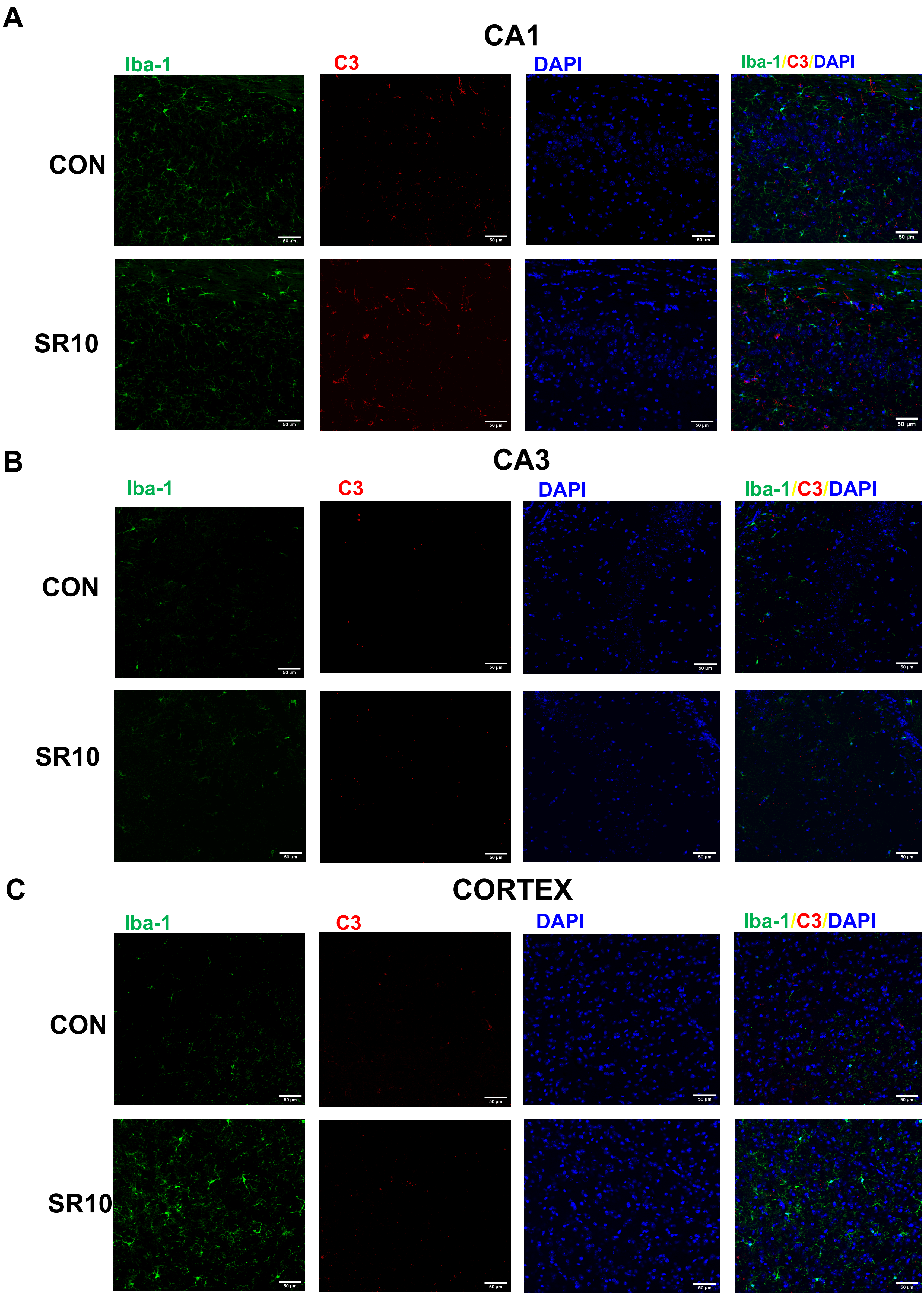
A
CA1
Iba-1
C3
DAPI
Iba-1/C3/DAPI
CON
SR10
B
CA3
Iba-1
C3
DAPI
Iba-1/C3/DAPI
CON
SR10
C
CORTEX
Iba-1
C3
DAPI
Iba-1/C3/DAPI
CON
SR10
50 μm

Fig. S2| C3 complement protein is not colocalized with microglia in the chronic sleep restriction model. Representative images of Iba-1+C3 expression in the hippocampal regions (A) CA1, (B) CA3, and in (C) cerebral cortex from control (CON) or 10-day sleep-restricted (SR10) mice are shown. Merge and each label of Iba-1 (green), C3 (red) and DAPI (blue) are shown separately.

Fig. S3

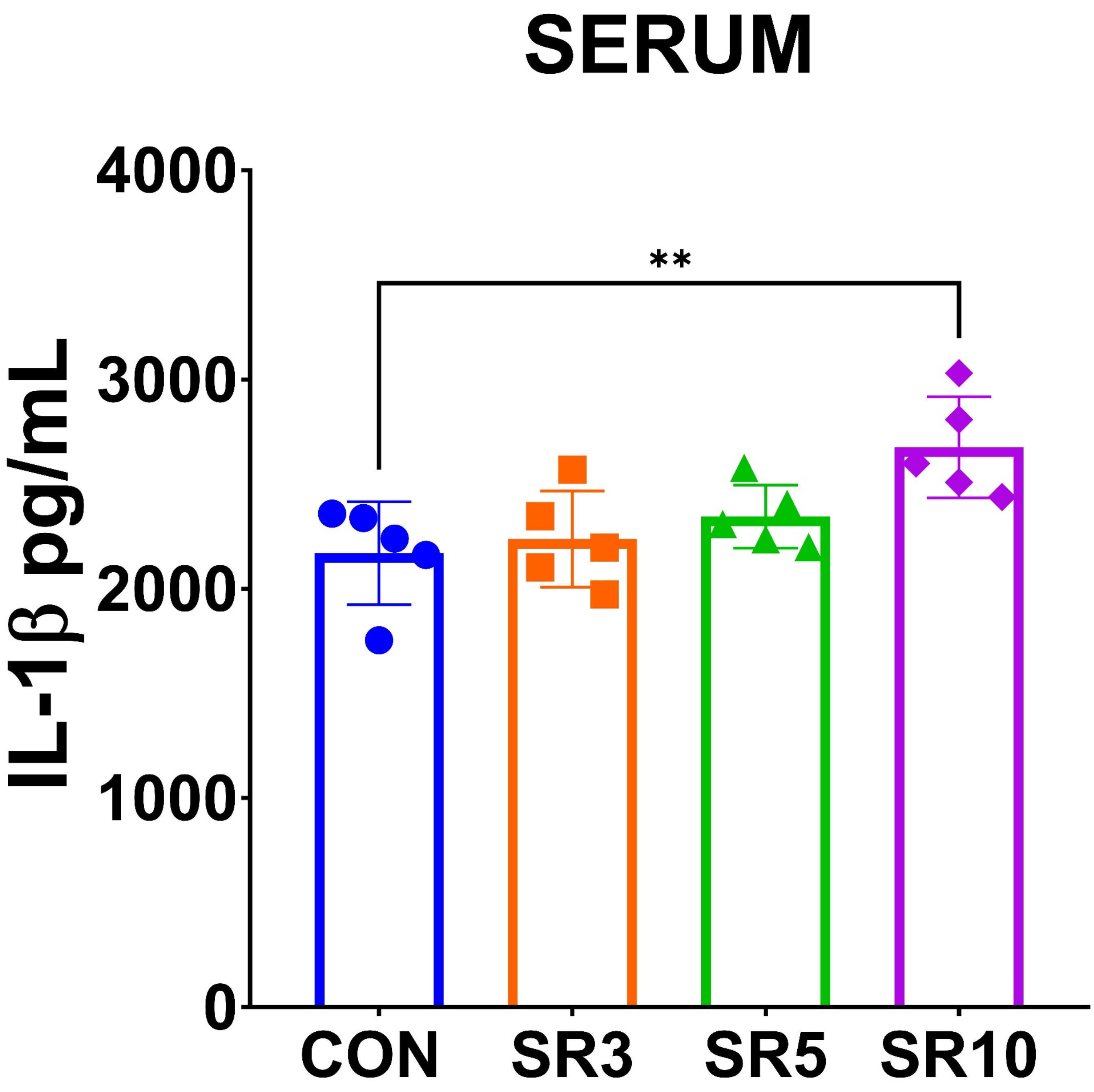

Fig. 6 Sleep restriction induces neuroinflammation. Graphs show the levels of pro-inflammatory cytokine IL-1β in the plasma of 3 (SR3), 5 (SR5), and 10 (SR10) days sleep-restricted mice, compared to intact controls (CON). Samples were quantified by duplicate (n = 5 per group). One-way ANOVA + post hoc Dunnett's test were performed. Mean ± standard deviation, *p < 0.05, **p < 0.01, ***p <0.001 compared to the CON.
